# Genetic‐based dissection of arsenic accumulation in maize using a genome‐wide association analysis method

**DOI:** 10.1111/pbi.12853

**Published:** 2017-12-04

**Authors:** Zhan Zhao, Huaisheng Zhang, Zhongjun Fu, Hao Chen, Yanan Lin, Pengshuai Yan, Weihua Li, Huiling Xie, Zhanyong Guo, Xuehai Zhang, Jihua Tang

**Affiliations:** ^1^ Key Laboratory of Wheat and Maize Crops Science Collaborative Innovation Center of Henan Grain Crops College of Agronomy Henan Agricultural University Zhengzhou China; ^2^ Maize Research Institute Chongqing Academy of Agricultural Sciences Chongqing China; ^3^ Hubei Collaborative Innovation Center for Grain Industry Yangtze University Jingzhou China

**Keywords:** arsenic accumulation, maize tissues, genetic loci, genome‐wide association analysis

## Abstract

Understanding the mechanism of arsenic (As) accumulation in plants is important in reducing As's toxicity to plants and its potential risks to human health. Here, we performed a genome‐wide association study to dissect the genetic basis of the As contents of different maize tissues in Xixian, which was irrigated with As‐rich surface water, and Changge using an association population consisting of 230 representative maize inbred lines. Phenotypic data revealed a wide normal distribution and high repeatability for the As contents in maize tissues. The As concentrations in maize tissues followed the same trend in the two locations: kernels < axes < stems < bracts < leaves. In total, 15, 16 and 15 non‐redundant quantitative trait loci (QTLs) associated with As concentrations were identified (*P* ≤ 2.04 × 10^−6^) in five tissues from Xixian, Changge, and the combination of the locations, respectively, explaining 9.70%–24.65% of the phenotypic variation for each QTL, on average. Additionally, four QTLs [involving 15 single nucleotide polymorphisms (SNPs)] were detected in the single and the combined locations, indicating that these loci/SNPs might be stable across different environments. The candidate genes associated with these four loci were predicted. In addition, four non‐redundant QTLs (6 SNPs), including a QTL that was detected in multiple locations according to the genome‐wide association study, were found to co‐localize with four previously reported QTL intervals. These results are valuable to understand the genetic architecture of As mechanism in maize and facilitate the genetic improvement of varieties without As toxicity.

## Introduction

Soil heavy metal and metalloid pollution have become a worldwide environmental problem owing to human activities, such as metal processing, mining, sewage, irrigation and applications of herbicides and fertilizers (Dai *et al*., 2016). Arsenic (As), which is a toxic metalloid, has been classified as a Group I carcinogen (Smith *et al*., 2002; Wu *et al*., 2015). Wilson *et al*. (2010) reported that the background concentration of As in soil is less than 10 mg/kg, while in some mining‐polluted soils, the As content is as high as 17 400 mg/kg. Because micronutrients in the soil are absorbed and accumulated by plants and enter the human body through the food chain (Dai *et al*., 2016), high As levels could cause serious human health risks. Sharma *et al*. (2016) reported that the consumption of As‐contaminated wheat grains by adults and children results in increased risks of developing cancer and noncancerous health disorders. And the consumption of rice grown in soils contaminated by As and other heavy metals near mine areas can also seriously affect heavy metal levels in human blood (Cai *et al*., 2015). Additionally, a high soil As level has many negative impacts on plant growth, causing damage to the plant's lateral roots and inhibiting water transport (Rahman *et al*., 2015; Zanella *et al*., 2016), thereby reducing crop quality and yield (Dai *et al*., 2016). It also negatively affects important plant metabolism‐associated processes, such as photosynthesis, transpiration and respiration, chlorophyll biosynthesis and nucleic acid synthesis, which results in hampered plant growth (Armendariz *et al*., 2016; Mishra *et al*., 2016; Rahman *et al*., 2015).

Considering the seriousness of the As‐associated damage to plant growth and the potential risks to human health, it is necessary to investigate the genetic mechanisms of As accumulation and tolerance in plants to reduce the toxicity. Quantitative trait locus (QTL) associated with As accumulation and tolerance in rice has been detected using different populations in previous study (Dasgupta *et al*., 2004; Zhang *et al*., 2008). Additionally, various genes involved in As accumulation in different species have been reported, such as, in tobacco, the overexpression of the *Arabidopsis thaliana*'s phytochelatin synthase 1 gene increases root As accumulation (Verma *et al*., 2016). The overexpression of the two As‐responsive rice glutaredoxin genes can reduce intracellular As accumulation and increase tolerance in *A. thaliana*, and significantly reduces arsenite accumulation by maintaining a glutathione pool and modulating aquaporins in yeast (Duan *et al*., 2016; Yang *et al*., 2016). In rice, the gene encoding a CRT‐like transporter is important for glutathione homeostasis and As tolerance (Yang *et al*., 2016). Yu *et al*. (2016) reported *A. thaliana*'s inositol transporter genes could increase As accumulation in *Saccharomyces cerevisiae* and regulated As accumulation in *A. thaliana* seeds.

The genome‐wide association analysis (GWAS), as a powerful tool, has been widely used to detect genetic variants related to complex quantitative traits in different plants (Atwell *et al*., 2010; Huang *et al*., 2010), such as the salt tolerance during germination in autotetraploid alfalfa (Norton *et al*., 2014), drought‐related metabolic changes in maize (Zhang *et al*., 2016), drought tolerance in maize seedlings (Wang *et al*., 2016) and heavy metal tolerance in rice (Norton *et al*., 2014). In a recent report, six candidate genes with pleiotropic effects on stalk’ cell wall components in maize have been identified by main of GWAS method (Li *et al*., 2016a). And in *A. thaliana,* a gene associated with leaf arsenic accumulation was identified through GWAS (*HAC1*, Chao *et al*., 2014) and linkage mapping (*ATQ1,* Sanchez‐Bermejo *et al*., 2014), respectively, the gene encoded a protein that as an arsenate reductase playing a critical role in arsenate resistance.

Maize, as an important crop over the world, is also a model plant. In the genetic dissection of As concentration in previous study, Liu *et al*. (2012) investigated trend in As accumulation and distribution in the different tissues of the 122 elite inbred maize lines. Ding *et al*. (2011) and Fu *et al*. (2016) used linkage mapping to unravel the genetic basis of As accumulation in two maize recombinant inbred line populations and found that As accumulation in different tissues in maize has a different molecular mechanism. However, the genetic basis of As concentration in maize is unclear up to now. Here, a GWAS with high‐density single nucleotide polymorphisms (SNPs) was used to identify the natural allelic variations that contribute to As accumulation in maize tissues, such as kernels, axes, stems, bracts and leaves. The main purposes were as follows: (i) to explore the rules of As accumulation and distribution in different maize tissues of an association population; and (ii) to investigate the significant SNPs/loci and potential candidate genes associated with As variation in the tissues of maize under different soil As concentration. This research will further our understanding of the mechanisms of As accumulation and aid in the development of strategies to ensure food safety.

## Results

### Performance of the measured traits

**Figure 1:**
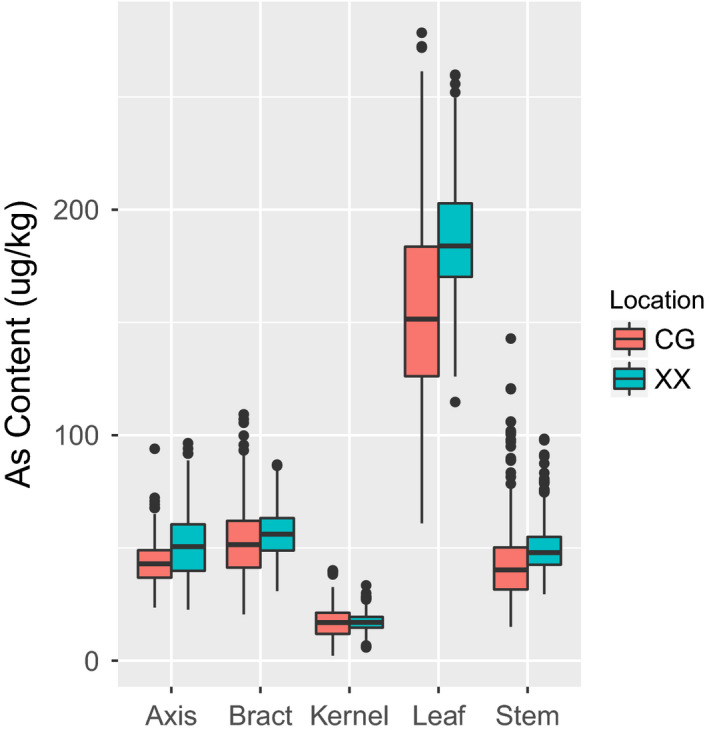
Distribution of the arsenic content measured in maize association mapping populations (AMPs). Box plots showing the arsenic contents among the AMPs. Data from two locations, Changge (CG) and Xixian (XX), are shown.

In the association population, the As contents in five maize tissues were higher from the Xixian (XX) location compared with the corresponding tissues from the Changge (CG) location. This might be attributed to a higher content of As in soil at XX than that at CG, and it suggested that soil As is an important determinant affecting the As content in maize tissues. The average As contents in kernels, axes, stems, bracts and leaves were 17.07, 51.22, 50.44, 56.61 and 186.65 μg/kg, at XX location, and 16.69, 43.48, 44.48, 53.08 and 157.15 μg/kg, respectively, at CG location. Combining data from the two locations, the average As contents in kernels, axes, stems, bracts and leaves were 16.88, 47.35, 47.46, 54.84 and 171.90 μg/kg, respectively (Table 1). In the association population, the As concentrations varied widely in all five tissues independent of the location and in the combined data (Figure 1, Figure S1). For example, the As contents of the combined environment in kernels, axes, stems, bracts and leaves ranged from 5.92 to 34.10 μg/kg, 27.27 to 88.86 μg/kg, 26.25 to 110.72 μg/kg, 31.06 to 91.45 μg/kg and 115.05 to 247.67 μg/kg, respectively (Table 1). The frequency of the As content's phenotypic value for data combined from the two locations exhibited an approximately normal distribution (Figure S1). For the As concentrations in different maize tissues, the same trend, kernels < axes < stems < bracts < leaves (Figure 1), was consistent with a previous report (Fu *et al*., 2016). Moreover, the Pearson correlation coefficient showed that close relationships were observed among the As concentrations between same tissues across locations (Table S1). According to the variance analysis (Table 2), the As contents in the five tissues in plants of the association population showed significant variations in environment, genotype and the genotype × environment interaction. The repeatability levels for the As contents in kernels, axes, stems, bracts and leaves were 86.73%, 86.71%, 86.46%, 86.79% and 82.16%, respectively (Table 2).

**Table 1:** Descriptive statistics for the arsenic contents in different maize tissues in the association population

Location	Trait	Mean ± SD^a^ (μg/kg)	Range (μg/kg)	Kurt^b^	Skew^c^
Xixian	Kernel	17.07 ± 3.66	7.52–31.02	1.04	0.31
	Axis	51.22 ± 14.13	24.96–92.80	−0.09	0.4
	Stem	50.44 ± 9.53	33.86–88.38	2.63	1.44
	Bract	56.61 ± 8.40	35.80–81.14	−0.05	0.29
	Leaf	186.65 ± 17.98	139.60–234.57	0.09	0.32
Changge	Kernel	16.69 ± 7.32	2.59–39.46	0.48	0.39
	Axis	43.48 ± 7.58	27.78–83.30	3.02	1.02
	Stem	44.48 ± 19.78	16.97–136.36	3.28	1.57
	Bract	53.08 ± 16.47	21.75–107.12	0.68	0.67
	Leaf	157.15 ± 42.77	63.52–275.18	−0.18	0.47
BLUP	Kernel	16.88 ± 4.98	5.92–34.10	0.74	0.38
	Axis	47.35 ± 10.45	27.27–88.86	0.61	0.57
	Stem	47.46 ± 13.98	26.25–110.72	3.31	1.59
	Bract	54.84 ± 11.56	31.06–91.45	0.44	0.55
	Leaf	171.90 ± 27.74	115.05–247.67	−0.01	0.54

a SD, Standard Deviation.

b Kurt, kurtosis, which is a measure of the ‘tailedness’ of the probability distribution of a real‐valued random variable.

c Skew, skewness, which is a measure of the asymmetry of the probability distribution of a real‐valued random variable about its mean.

**Table 2:** Variance analysis of the five measured tissues across two locations in the association population

Tissue	Variance	MS	*F*	*P* value^c^	*w* ^2^ (%)^d^
Kernel	E	51.11	7.86^b^	5.15E‐03	86.73
	G	198.97	30.6^a^	2.00E‐16	
	G*E	26.42	4.06^a^	2.00E‐16	
Axis	E	20708.39	350.36^a^	1.72E‐66	86.71
	G	874.92	14.8^a^	1.65E‐198	
	G*E	116.24	1.97^a^	2.37E‐12	
Stem	E	12283.58	118.67^a^	4.46E‐26	86.46
	G	1575.83	15.22^a^	1.20E‐202	
	G*E	213.33	2.06^a^	5.88E‐14	
Bract	E	4286.86	83.83^a^	3.41E‐19	86.79
	G	1068.87	20.9^a^	1.97E‐251	
	G*E	141.18	2.76^a^	8.87E‐27	
Leaf	E	300372.56	516.69^a^	3.95E‐91	82.16
	G	6871.67	11.82^a^	1.27E‐166	
	G*E	1225.78	2.11^a^	8.80E‐15	

a significant at α = 0.001.

b significant at α = 0.01; E: environment, G: genotype, G × E: genotype‐by‐environment interaction.

c *P* value, statistical significance of five measured tissues in the two locations.

d *w* ^2^, repeatability.

### GWAS

Several genotyping platforms (Illumina MaizeSNP50 BeadChip, RNA sequencing, genotyping by sequencing and Affymetrix Axiom Maize 600K array) that integrated effective imputation methods and generated 1.25 M SNPs with a minor allele frequency greater than 0.05 were used in this study (Liu *et al*., 2017). Three models, Q (only accounts for population structure), K (only accounts for relative kinship) and Q + K (accounts for population structure and relative kinship), were employed to perform GWAS for the As concentrations in five maize tissues at two separate locations and a combination of the two locations. To test the optimal GWAS model, quantile–quantile plots (QQ plots) for each trait under the three models were plotted (Figure S2), and the QQ plots indicated that the Q + K model was more reliable than the K model. However, the K and Q + K models were too strict in reducing the type I errors (false positives), which results in more type II errors (false negatives). The Q model's performance significantly reduced the type I errors (false positive) compared to the other two models. Thus, in the following analysis, the GWAS results from Q model will be further elucidated.

**Figure 2:**
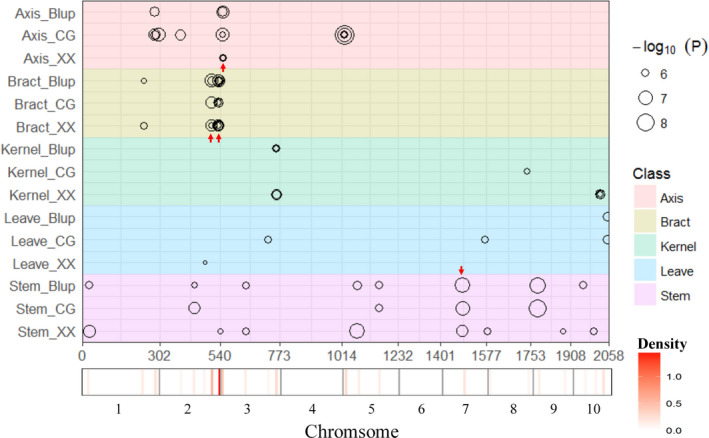
Chromosomal distribution of arsenic content‐associated QTLs identified in the maize association population. QTL position and significance (represented by circle size) across the maize genome responsible are shown as black circles. The *x*‐axis indicates the physical positions across the maize genome in Mb. The heat map under the *x*‐axis illustrates the density of QTLs across the genome. The window size is 10 Mb. Detailed information for all detected QTLs is shown in Table S2. Different traits are marked by distinct colours as shown on the right. Non‐redundant QTLs, which were detected simultaneously in XX, CG and the combination of the locations, are marked by red arrow.

**Figure 3:**
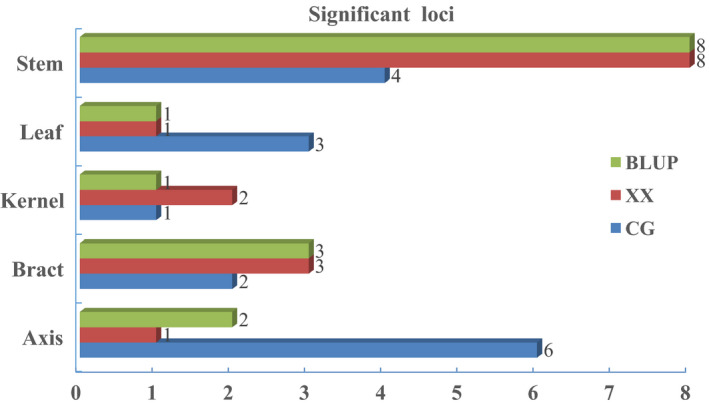
Comparison of significant association loci for five maize tissues from plants at different locations. Significant association loci for BLUP, Xixian (XX) and Changge (CG) are shown in green, red and blue, respectively.

According to the linkage disequilibrium (LD) decay of this association population (Liu *et al*., 2017), the 30‐kb regions flanking the left and right sides of each significant SNP could be defined as QTLs. Under the Q model, 79 SNP‐trait associations were detected at the significance level of *P* ≤ 2.04E‐06 in five tissues (Table S2), which involving 45 SNPs. According to the definition of the QTLs, 45 QTLs were detected in total. Furthermore, these QTLs can be categorized into 28 non‐redundant QTLs (all of the QTLs with overlapping flanking intervals were categorized as non‐redundant QTLs). Briefly, in XX, 15 non‐redundant QTLs (27 significant SNPs) were identified in five tissues, and each locus could explain phenotypic variation (*R* ^2^) ranging from 9.20% to 18.74% and a mean of 11.59%. In CG, 16 non‐redundant QTLs (25 significant SNPs) were detected in five tissues, and each locus could explain phenotypic variation ranging from 9.70% to 24.65%, with a mean of 12.67%. Moreover, 15 non‐redundant QTLs (27 significant SNPs) were identified in the combination of the two locations, with an *R* ^2^ ranging from 9.70 to 19.70%, and a mean of 11.87% (Figures 2 and 3, Table 3, Table S2). Furthermore, the same 27 significant SNPs from the GWAS of BLUP data could still be identified in the GWAS of LSM date, and only the *P* value and *R* ^2^ of each SNP has slight difference (Table S3). The detailed information for the GWAS results, including *P* value and *R* ^2^ of each non‐redundant QTL, physical positions of peak SNPs and the most likely candidate genes and their annotations, is listed in Table S2. Manhattan plots for As concentrations in all five tissues at XX, CG and the combination are shown in Figure S3. All potential candidate genes and their functional annotations within 60 kb (30‐kb upstream and downstream of the peak SNPs) of each non‐redundant QTL identified from GWAS are provided in Table S2.

**Table 3:** Summary of significant loci–trait associations identified by the genome‐wide association study

	CG^a^	XX	BLUP
Number of traits^b^	5/5	5/5	5/5
Number of loci^c^	16	15	15
Average loci per trait^d^	3.20 ± 1.72	3.00 ± 2.61	3.00 ± 2.61

a CG and XX represent the two locations Changge and Xixian, respectively; BLUP, Best linear unbiased prediction.

b Number of traits having significantly associated loci/number of total detected traits.

c Number of significant loci detected in each location on the association panel [*P* ≤ 2.04 × 10^−6^, Q model].

d Average number of significant loci (or QTLs) detected per trait ± SD.

In Table 4, the co‐localization of non‐redundant QTLs identified across different locations is summarized. Overall, 38 SNP‐trait associations, involving 15 significant SNPs in four non‐redundant QTLs and corresponding to three tissues (axis, bract and stem), were detected simultaneously in XX, CG and the combination of the locations (Figure 2). Thus, these QTLs might be stable across different environments. Furthermore, a QTL hot spot existed at the end of chromosome 2 (230.49–230.55 Mb) (Figure 2), indicating that this hot spot could be the key factor for regulating As accumulation in maize bracts. Detailed analyses of the candidate genes underlying these four non‐redundant QTLs will almost certainly provide useful information concerning As accumulation.

**Table 4:** Candidate genes revealed by multiple locations

ID^a^	Loci^b^	Trait	Location	Peak SNP	Chr.	Position (bp)^c^	QTL interval (Mb)^d^	*P* value^e^	*R* ^2^(%)^f^	Candidate gene^g^	Annotation^h^
17	9	Bract	BLUP	chr2.S_203601399	2	203 601 399	203.57–203.63	1.13E‐07	11.20	GRMZM2G125495	Extracellular‐glutamate‐gated ion channel activity
29	9	Bract	CG	chr2.S_203601399	2	203 601 399	203.57–203.63	3.56E‐07	10.39	GRMZM2G125495	Extracellular‐glutamate‐gated ion channel activity
37	9	Bract	XX	chr2.S_203601399	2	203 601 399	203.57–203.63	2.64E‐07	10.60	GRMZM2G125495	Extracellular‐glutamate‐gated ion channel activity
18	9	Bract	BLUP	chr2.S_203601456	2	203 601 456	203.57–203.63	1.13E‐07	11.20	GRMZM2G125495	Extracellular‐glutamate‐gated ion channel activity
30	9	Bract	CG	chr2.S_203601456	2	203 601 456	203.57–203.63	3.56E‐07	10.39	GRMZM2G125495	Extracellular‐glutamate‐gated ion channel activity
38	9	Bract	XX	chr2.S_203601456	2	203 601 456	203.57–203.63	2.64E‐07	10.60	GRMZM2G125495	Extracellular‐glutamate‐gated ion channel activity
19	9	Bract	BLUP	chr2.S_203602088	2	203 602 088	203.57–203.63	9.09E‐07	9.64	GRMZM2G125495	Extracellular‐glutamate‐gated ion channel activity
39	9	Bract	XX	chr2.S_203602088	2	203 602 088	203.57–203.63	1.53E‐06	9.28	GRMZM2G125495	Extracellular‐glutamate‐gated ion channel activity
20	10	Bract	BLUP	chr2.S_230520093	2	230 520 093	230.49–230.55	1.22E‐06	9.43	GRMZM2G052457	Adenosylmethionine‐8‐amino‐7‐oxononanoate transaminases
40	10	Bract	XX	chr2.S_230520093	2	230 520 093	230.49–230.55	1.65E‐06	9.20	GRMZM2G052457	Adenosylmethionine‐8‐amino‐7‐oxononanoate transaminases
21	10	Bract	BLUP	chr2.S_230520234	2	230 520 234	230.49–230.55	8.72E‐07	9.85	GRMZM2G052457	Adenosylmethionine‐8‐amino‐7‐oxononanoate transaminases
41	10	Bract	XX	chr2.S_230520234	2	230 520 234	230.49–230.55	9.56E‐07	9.76	GRMZM2G052457	Adenosylmethionine‐8‐amino‐7‐oxononanoate transaminases
22	10	Bract	BLUP	chr2.S_230520341	2	230520 341	230.49–230.55	3.38E‐07	10.50	GRMZM2G052457	Adenosylmethionine‐8‐amino‐7‐oxononanoate transaminases
31	10	Bract	CG	chr2.S_230520341	2	230 520 341	230.49–230.55	1.49E‐06	9.43	GRMZM2G052457	Adenosylmethionine‐8‐amino‐7‐oxononanoate transaminases
42	10	Bract	XX	chr2.S_230520341	2	230 520 341	230.49–230.55	3.38E‐07	10.51	GRMZM2G052457	adenosylmethionine‐8‐amino‐7‐oxononanoate transaminases
23	10	Bract	BLUP	chr2.S_230521739	2	230 521 739	230.49–230.55	1.08E‐06	9.68	GRMZM2G052457	Adenosylmethionine‐8‐amino‐7‐oxononanoate transaminases
24	10	Bract	BLUP	chr2.S_230521763	2	230 521 763	230.49–230.55	2.48E‐07	10.77	GRMZM2G052457	Adenosylmethionine‐8‐amino‐7‐oxononanoate transaminases
32	10	Bract	CG	chr2.S_230521763	2	230 521 763	230.49–230.55	6.78E‐07	10.06	GRMZM2G052457	Adenosylmethionine‐8‐amino‐7‐oxononanoate transaminases
43	10	Bract	XX	chr2.S_230521763	2	230 521 763	230.49–230.55	5.87E‐07	10.14	GRMZM2G052457	Adenosylmethionine‐8‐amino‐7‐oxononanoate transaminases
25	10	Bract	BLUP	chr2.S_230522102	2	230 522 102	230.49–230.55	2.69E‐07	10.71	GRMZM2G052457	Adenosylmethionine‐8‐amino‐7‐oxononanoate transaminases
33	10	Bract	CG	chr2.S_230522102	2	230 522 102	230.49–230.55	8.50E‐07	9.89	GRMZM2G052457	Adenosylmethionine‐8‐amino‐7‐oxononanoate transaminases
44	10	Bract	XX	chr2.S_230522102	2	230 522 102	230.49–230.55	4.92E‐07	10.27	GRMZM2G052457	Adenosylmethionine‐8‐amino‐7‐oxononanoate transaminases
26	10	Bract	BLUP	chr2.S_230523364	2	230 523 364	230.49–230.55	2.46E‐07	10.82	GRMZM2G052457	Adenosylmethionine‐8‐amino‐7‐oxononanoate transaminases
34	10	Bract	CG	chr2.S_230523364	2	230 523 364	230.49–230.55	8.55E‐07	9.92	GRMZM2G052457	Adenosylmethionine‐8‐amino‐7‐oxononanoate transaminases
45	10	Bract	XX	chr2.S_230523364	2	230 523 364	230.49–230.55	3.82E‐07	10.50	GRMZM2G052457	Adenosylmethionine‐8‐amino‐7‐oxononanoate transaminases
27	10	Bract	BLUP	chr2.S_230523621	2	230 523 621	230.49–230.55	1.43E‐06	9.81	GRMZM2G052457	Adenosylmethionine‐8‐amino‐7‐oxononanoate transaminases
28	10	Bract	BLUP	chr2.S_230523716	2	230 523 716	230.49–230.55	2.46E‐07	10.82	GRMZM2G052457	Adenosylmethionine‐8‐amino‐7‐oxononanoate transaminases
35	10	Bract	CG	chr2.S_230523716	2	230 523 716	230.49–230.55	8.55E‐07	9.92	GRMZM2G052457	Adenosylmethionine‐8‐amino‐7‐oxononanoate transaminases
46	10	Bract	XX	chr2.S_230523716	2	230 523 716	230.49–230.55	3.82E‐07	10.50	GRMZM2G052457	Adenosylmethionine‐8‐amino‐7‐oxononanoate transaminases
2	12	Axis	BLUP	chr3.S_7675260	3	7 675 260	7.65–7.71	7.28E‐07	10.42	GRMZM5G820781	Unknown
8	12	Axis	CG	chr3.S_7675260	3	7 675 260	7.65–7.71	1.52E‐06	9.81	GRMZM5G820781	Unknown
14	12	Axis	XX	chr3.S_7675260	3	7 675 260	7.65–7.71	1.77E‐06	9.76	GRMZM5G820781	Unknown
3	12	Axis	BLUP	chr3.S_7683509	3	7 683 509	7.65–7.71	2.25E‐07	11.11	GRMZM5G820781	Unknown
9	12	Axis	CG	chr3.S_7683509	3	7 683 509	7.65–7.71	1.47E‐07	11.35	GRMZM5G820781	Unknown
15	12	Axis	XX	chr3.S_7683509	3	7 683 509	7.65–7.71	1.38E‐06	9.78	GRMZM5G820781	Unknown
65	19	Stem	BLUP	chr7.S_84124201	7	84 124 201	84.09–84.15	4.58E‐08	19.70	GRMZM2G057317	Unknown
70	19	Stem	CG	chr7.S_84124201	7	84 124 201	84.09–84.15	8.20E‐08	18.98	GRMZM2G057317	Unknown
76	19	Stem	XX	chr7.S_84124201	7	84 124 201	84.09–84.15	3.23E‐07	17.50	GRMZM2G057317	Unknown

a The ID of the SNP/trait associations and non‐redundant QTLs.

b Loci identified in the GWAS (correspond to those in Table S2).

c Physical position in base pairs for the peak SNP according to version 5b.60 of the maize reference sequence.

d 30‐kb upstream and downstream of the peak SNP.

e *P*‐values of peak SNPs estimated by the Q model.

f The phenotypic variance explained by the corresponding locus.

g Plausible biological candidate genes in the physical intervals of each QTL.

h Annotation information according to InterProScan (http://www.ebi.ac.uk/interpro/).

### Candidate genes revealed by multiple locations

Based on the GWAS, 28 non‐redundant QTLs were identified in this study (Table S2). This was consistent with the quantitative nature of As content in maize being controlled by a large number of QTLs, as well as with previously studied agronomic traits (Ding *et al*., 2011; Fu *et al*., 2016; Xiao *et al*., 2016). Interestingly, four non‐redundant QTLs that were detected simultaneously in both single‐environment analysis and the joint analysis were used to identify their candidate genes based on LD decay or supporting flanking intervals. A total of nine candidate genes were found (Table S2), including five candidate genes (*GRMZM2G125487*, *GRMZM2G125527*, *GRMZM2G125495*, *GRMZM2G426556* and *GRMZM2G125507*) located in the 9th non‐redundant QTL (from 203.57 Mb to 203.63) on chromosome 2 that were significantly associated with the As content in the bracts. *GRMZM2G125495* encodes a protein that participates in extracellular‐glutamate‐gated ion channel activity and is a likely candidate for the gene that transports As ions in plants. Only one gene, *GRMZM5G820781*, located in the 12th non‐redundant QTL (from 7.65 Mb to 7.71 Mb) on chromosome 3 was significantly associated with the As content in the axes. The gene *GRMZM5G820781* encodes an uncharacterized protein, which was only expressed in pericarp aleurone. Additionally, no genes in the 19th non‐redundant QTL (from 84.09 Mb to 84.15 Mb) on chromosome 7 significantly associated with the As content in the stem, while *GRMZM2G057317*, which is nearest to the peak SNP, encodes an unknown protein that was only expressed in the embryo. Moreover, three candidate genes (*GRMZM2G052457*, *GRMZM2G158872* and *GRMZM2G452669*) located in the 10th non‐redundant QTL on chromosome 2 (230.49–230.55 Mb) were significantly associated with the As content in the bracts. These three genes also formed the only QTL hot spot identified in this study. *GRMZM2G052457* encodes an adenosylmethionine‐8‐amino‐7‐oxononanoate transaminases, *GRMZM2G158872* has an unknown function, and *GRMZM2G452669* encodes an alpha‐galactosidase precursor. The most likely candidate genes are listed in Table 4.

## Discussion

When using a GWAS, the power, that is the probability of detecting the causal variant, should be the first consideration. Additionally, traits are sensitive to different statistical models, which vary in their abilities to control type I or type II errors. Thus, the models can be seen as trait‐dependent. For example, Yang *et al*. (2011) reported that the K and Q + K models performed well for flowing time, ear height and ear diameter; however, the Q + K models performed slightly better than the K model for all three traits (Yang *et al*., 2011). In the present study, the Q model, which was selected to illustrate the GWAS results, had a greater ability to correct false‐positive associations than the K and Q + K models (Figure S2), although it could not completely control the population structure.

GWAS‐based mapping has been successfully used to identify a new arsenate reductase enzyme critical for limiting As accumulation in *A. thaliana* (Chao *et al*., 2014). In this study, only 15 SNPs or 4 QTLs, which stably existed across different environments and significantly associated with As accumulation in different maize tissues, were identified through a GWAS, implying that As accumulation in different maize tissues may be controlled by different genetic mechanisms. However, the results will be helpful in illustrating the genetic mechanisms and cloning the genes governing As accumulation in maize. In *A. thaliana*, GWAS and QTL mapping are used to complement each other, overcoming their individual limitations (Tiwari *et al*., 2016). In maize, the combination of QTL mapping and GWAS has been used to identify candidate genes for plant ear height and ear traits (Li *et al*., 2016b; Xiao *et al*., 2016). The combination of the two methods has also been applied to detect loci governing agronomic traits in other species, like soya bean (Zhao *et al*., 2015) and rice (Lou *et al*., 2015).

In this study, six SNPs associated with four non‐redundant QTLs that significantly associated with As accumulation, as detected by GWAS, were found to co‐localize (here, any two QTLs with physical positions less than 5 Mb apart were declared as co‐localized) with a previously detected QTL from a linkage population (Fu *et al*., 2016; Table S2). The loci, located from 25.71 to 25.77 Mb on chromosome 1, co‐localized with the previously reported QTL *CAsA1/CAsS1*, and only one candidate gene, *GRMZM2G130987*, was found. The gene encodes a protein that has P‐P‐bond‐hydrolysis‐driven protein transmembrane transporter activity, which may participate in As ion transport. The other three significant SNPs (chr2.S_203601399, chr2.S_203601456 and chr2.S_203602088) on chromosome 2 were found within the QTL *BAsA2*/*XAsA2*. This QTL was also detected in multiple locations in our study. Five candidate genes (*GRMZM2G125487*, *GRMZM2G125527*, *GRMZM2G125495*, *GRMZM2G426556* and *GRMZM2G125507*) are located in this QTL. *GRMZM2G125495* encodes a protein that has extracellular‐glutamate‐gated ion channel activity and is a likely candidate gene. This provides a solid reason to further study the gene's function in As accumulation. Another region, between 90.45 Mb and 90.51 Mb on chromosome 10, contained one SNP (chr10.S_90481204), which co‐localized with the QTL *XAsA10* that includes four candidate genes (*GRMZM2G166665*, *GRMZM2G166616*, *GRMZM2G166639* and *GRMZM2G166694*). On chromosome 10, the region from 143.13 to 143.19 Mb, which contains three genes (*GRMZM2G326066*, *GRMZM2G021822* and *GRMZM2G021885*), was co‐localized with *CAsL10*. The joint use of linkage mapping and GWAS was more efficient in identifying candidate genes and detecting loci governing As accumulation in maize tissues. These co‐localized QTLs from different genetic populations and the common QTLs were all detected in the same population at multiple locations, which will provide useful reference information for studies on the functional verification of As accumulation and for breeding varieties with low As concentrations.

The highest As toxicity in plants was primarily attributed to two aspects. First, a high As level can reduce phosphate uptake from the agricultural soil because of their structural similarities. Second, the presence of As (V) in plants could lead to oxidative stress by altering the synthesis of adenosine triphosphate and the phosphate group of DNA (Rosas‐Castor *et al*., 2014). In a previous study, the induction of oxidative stress was reported as the main process underlying As toxicity in plants and the up‐regulation of a set of oxidative stress‐related proteins could induce the plant's responses to acute inorganic As toxicity. This regulation might be a consequence of the production of reactive oxygen species derived from As (Requejo and Tena, 2005). Furthermore, He *et al*. (2006) first proved that arsenic could induce NAD(P) H‐quinone oxidoreductase I by activating the Nrf2/Keap1 signalling pathway and recruiting Nrf2/Maf to the antioxidant response element enhancer. In the present study, most of the candidate genes were not annotated, perhaps because of the limited study of As accumulation in maize. However, two candidate genes, *GRMZM2G135044* and *GRMZM2G166616*, which were annotated, encode proteins that have oxidoreductase activities and may also be involved in responding to As exposure.

Heavy metal and metalloid pollution in soil is a serious problem threating human health. As could be transferred from food chain to human body, if humans long‐term exposure to inorganic arsenic will result in an increased incidence a range of cancers, particularly of the skin, lung, bladder and liver, as well as liver injury, cardiovascular disease, postnatal growth retardation and so on (Huang *et al*., 2015; Li *et al*., 2016c). Therefore, it is very important to control food source through breeding the crop varieties without As toxicity. However, for maize, during the As‐exposure period, there is a change in the nutritional state, especially in the contents of minerals elements, such as Mg, K, Ca and Fe (Rosas‐Castor *et al*., 2014). These minerals slowly ascend to the corn aerial parts, and As's effects on their levels remain unclear (Mallick *et al*., 2011). To systematically explore the relationship of these elements, thus, not just As, but other heavy metal ions, should be considered in future studies. High‐throughput methods, such as ion omics (Salt *et al*., 2008), are alternative ways to explore the relationships among these ions and to dissect the key loci governing these important indicators. This will lead to the more complete knowledge and understanding of the mechanisms involved in heavy metal ion accumulation, which will help increase food security.

## Experimental procedures

### Plant treatments and soil conditions

A representative sample of 230 inbred lines, with 151 from the temperate zone and 79 from tropical and subtropical zones, were used in the association population. The association population was planted in 2012 in two locations, Xinxian (XX, E114°72′, N32°35′) and Changge (CG, E113°34′, N34°09′) Counties, China, which are located in northern China, with average temperatures of 15.2 and 14.3 °C, and rainfalls of 873.8 and 462.8 mm, respectively. The soil As contents at these two locations were significantly different owing to irrigation with As‐rich surface water in XX. The soil As concentration at XX was 20.70 ± 0.37 mg/kg (pH 6.5), which was much greater than that at CG (12.24 ± 0.21 mg/kg, pH 6.5). At each location, the association population was laid out in a randomized complete block design, with three replications. Plots consisted of 4‐m rows that were 0.22 m apart, with 0.67‐m in‐row spacing. The final plant density was 67 500 plants per hectare.

### Determination of the As concentration in maize tissues

The association population was harvested to determine the accumulation and distribution of As in different maize tissues when they were physiologically mature. In each plot, the kernels, axes, stems, bracts and leaves from five consecutive plants were total collected together, respectively. After the tissues were dried, samples were used to determine the As concentration. Briefly, each dry tissue of five consecutive plants in each plot was first ground into a fine powder using a grinding mill. Then, through a heating block (AIM500 Digestion System, A.I. Scientific, Australia), the powdered samples (0.5 g) were digested with 5 mL of HNO_3_/HClO_4_ (80/20, v/v) in polypropylene tubes. The As concentration (μg/kg) was determined in the different maize tissues using atomic fluorescence spectrometry (AFS‐3000, Beijing Haiguang Analytical Instrument Co., Beijing, China). Each sample was measured three times, the average data of As concentration of each tissue were calculated first, and then, the average value of As concentration in each tissue for one material in the three replications was used to detect the significant loci/SNP. A two‐way analysis of variance was used to analyse the data using the IBM SPSS Statistics package, and repeatability was also calculated based on the method developed by Knapp (1986).

Repeatability (*w* ^2^) for each trait across the two locations was computed as follows:

$w^{2}=\sigma_{G}^{2}/\left[\sigma_{G}^{2}+(\sigma_{\mathrm{GE}}^{2}/n\right)+\sigma_{e}^{2}/\left(\text{nr}\right)]$.

In the above formula, $\sigma_{G}^{2}$ represents the genotypic variance, $\sigma_{\mathrm{GE}}^{2}$ represents the genotype × environment variance, $\sigma_{e}^{2}$ represents the error variance, n represents the number of locations, and r represents the number of replications. The estimates of $\sigma_{G}^{2}$, $\sigma_{\mathrm{GE}}^{2}$ and $\sigma_{e}^{2}$ were analysed by an analysis of variance using the lmer function in the lme4 package in the R environment (R Core Team, 2012; version 3.1.3; http://www.r-project.org/).

The best linear unbiased prediction (BLUP) was obtained by fitting the mixed linear model in the R package lem4 (R Core Team, 2012) for the estimation of the breeding values of each line across two locations, and the formula is

$$Y=\left(1|\text{LINE}\right)+\left(1|\text{ENV}\right)+\left(1|\text{REP}\%\text{in}\%\text{LINE:ENV}\right)+\left(1|\text{LINE:ENV}\right)$$

where *Y* is trait data, the parenthesis indicates the random effects, ‘1|’ means groups, and ‘:’ means interactions. LINE indicates all testcrosses used; ENV indicates the environments, each of which is a combination of years and locations; and REP is the replications in one ENV. And the BLUP values were then combined to reduce the prediction bias caused by the unbalanced data. Finally, the BLUP data for the As concentrations of each tissue across two locations were also used for the GWAS. The Pearson correlation coefficients among the As contents of different tissues were implemented with SPSS software (v13.0). To test whether the major QTLs from BLUP values still be found, LSM (least square means) of the data from different locations of the same tissue were calculated in the R package lsmeans (R Core Team, 2012). And the LSM values for the As concentrations of each tissue across two locations were also used for the GWAS.

### GWAS

According to the method described by Liu *et al*. (2017), 1.25 million (M) SNPs (1, 253, 814 SNPs) with a minor allele frequency ≥5% were obtained by combining genotypes from previous RNA sequencing (Fu *et al*., 2013) and the Illumina MaizeSNP50 BeadChip (Ganal *et al*., 2011) with newly identified SNPs from the Affymetrix Axiom Maize 600K array (Unterseer *et al*., 2014) and genotyping by sequencing technology (Elshire *et al*., 2011) was used for the GWAS (the SNP data are available at http://www.maizego.org/Resources.html).

A GWAS was conducted for five different tissues at XX, CG and the combination of the locations. To test the optimal GWAS model, three models, Q (only accounts for population structure), K (only accounts for relative kinship) and Q + K (accounts for population structure and relative kinship), were implemented in the software TASSEL 3.0 (Bradbury *et al*., 2007) to test the statistical associations between genotype and phenotype.

Because many of the SNPs should be in linkage disequilibrium, the effective number of independent markers was also calculated using the GEC software tool in a previous study (Deng *et al*., 2017), and the suggested *P*‐value of 2.04E‐06 (1/independent marker number) was used to control the genome‐wide type I error rate. The *P*‐value of each SNP was obtained from TASSEL 3.0 (Bradbury *et al*., 2007) and used to construct QQ and Manhattan plots for the As concentration in each tissue.

### Analysis of candidate genes

Based on the reported maize B73's genome sequence (RefGen_v2), the filtered maize working gene list was downloaded from MaizeGDB (http://www.maizegdb.org) and used to identify possible candidate genes in each QTL. Candidate genes were annotated according to InterProScan (http://www.ebi.ac.uk/interpro/scan.html). In the previous study, the LD of the association population was estimated using the 1.25 M SNPs and the LD decay was 30 kb (*r* ^2^ = 0.1) (Liu *et al*., 2017). All of the potential candidate genes and their annotations within 60 kb (30‐kb upstream and downstream of the peak SNP, which is the SNP with the lowest *P*‐value) of the identified loci are listed in Table S2. For the loci without appropriate candidates, the gene nearest the peak SNP was assigned. The physical locations of the SNPs were based on B73 RefGen_v2.

## Supporting information


**Figure S1** Frequency distribution of the As contents in five maize tissues from plants at the combined locations.
**Figure S2** Quantile–quantile plots constructed using genome‐wide association study results from three models (Q, K and Q+K) for the arsenic contents in five tissues across different locations.
**Figure S3** Manhattan plots for the arsenic contents in five different tissues across different locations.

**Table S1** Correlation coefficients among five maize tissues in the association population.

**Table S2** List of significant loci, their detailed information and all candidate genes within the significant loci identified by the GWAS.

**Table S3** Comparison of GWAS results using the BLUP data and least square means from different locations of the same tissue.

Supplementary Legends
