# Supplementary material for: Genetic‐based dissection of arsenic accumulation in maize using a genome‐wide association analysis method
Source: Plant Biotechnol J. 2017 Dec 4;16(5):1085–93. doi: 10.1111/pbi.12853 (PMC5902774; doi:10.1111/pbi.12853)
Supplement: Supplementary file 1 — Figure S1 Frequency distribution of the As contents in five maize tissues from plants at the combined locations. Figure S2 Quantile–quantile plots constructed using genome‐wide association study results from three models (Q, K and Q+K) for the arsenic contents in five tissues across different locations. Figure S3 Manhattan plots for the arsenic contents in five different tissues across different locations. [file PBI-16-1085-s004.docx]

SUPPLEMENTAL INFORMATION

This file includes Figure S1-S3. Table S1-S3 are separate excel files and are available at Plant Biotechnology Journal.


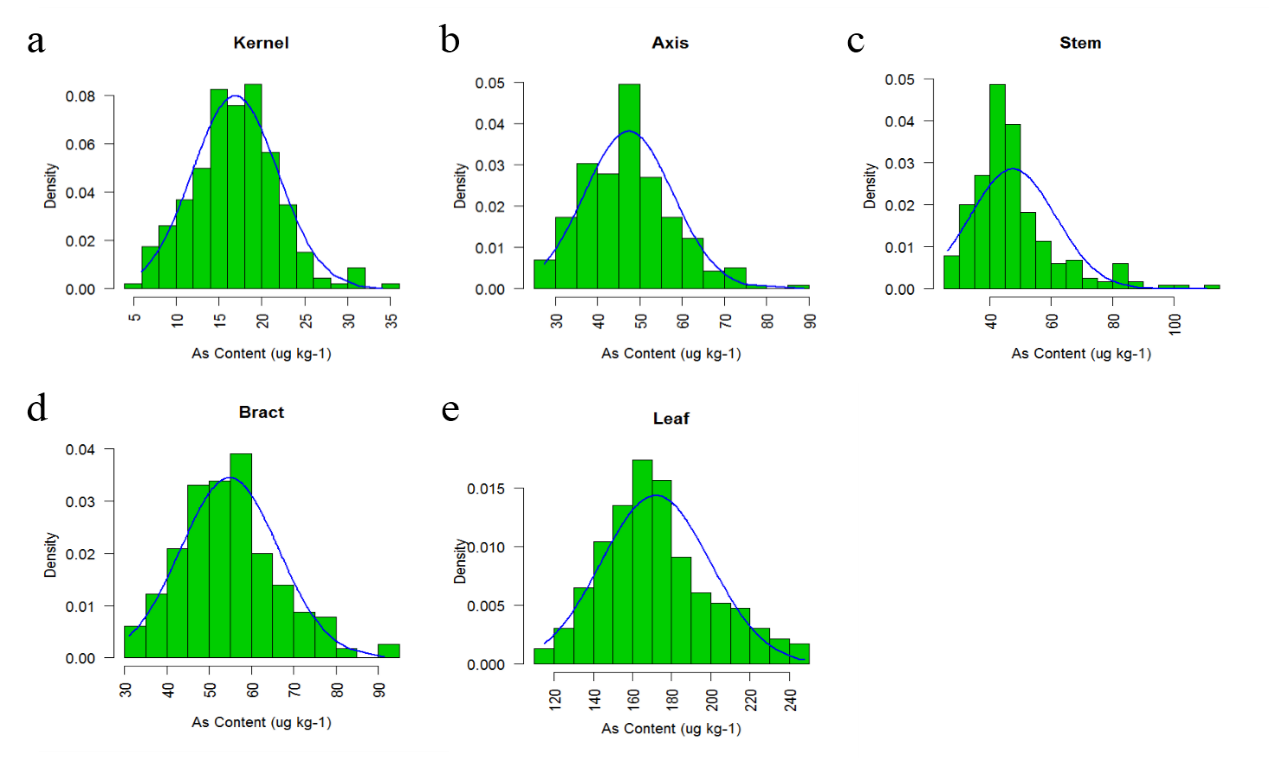


**Figure S1.** Histogram of the arsenic concentrations in five maize tissues combined from Changge (CG) and Xixian (XX) in the association population. a-e represents kernel, axis, stem, bract and leaf, respectively.


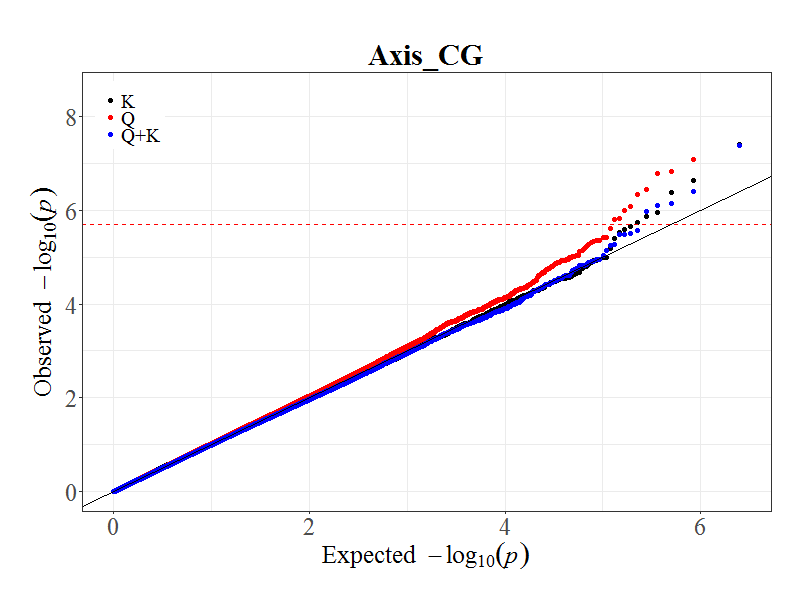

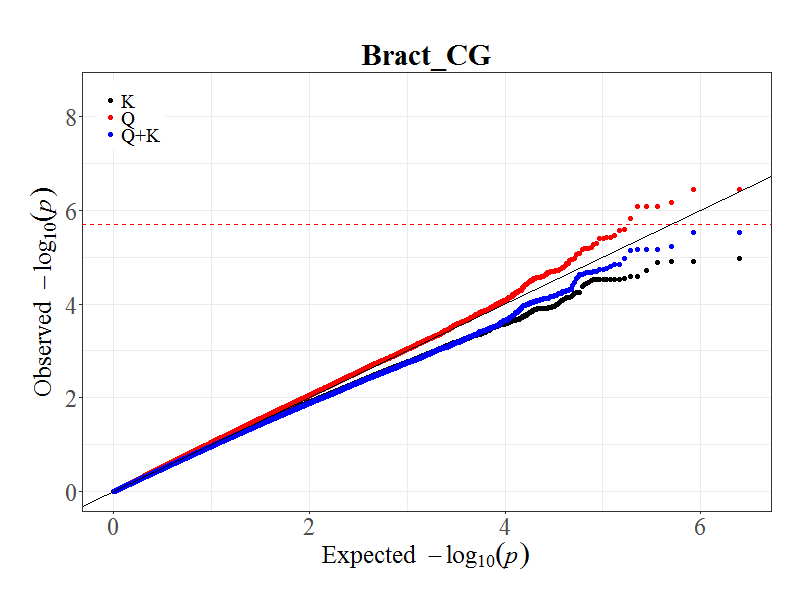

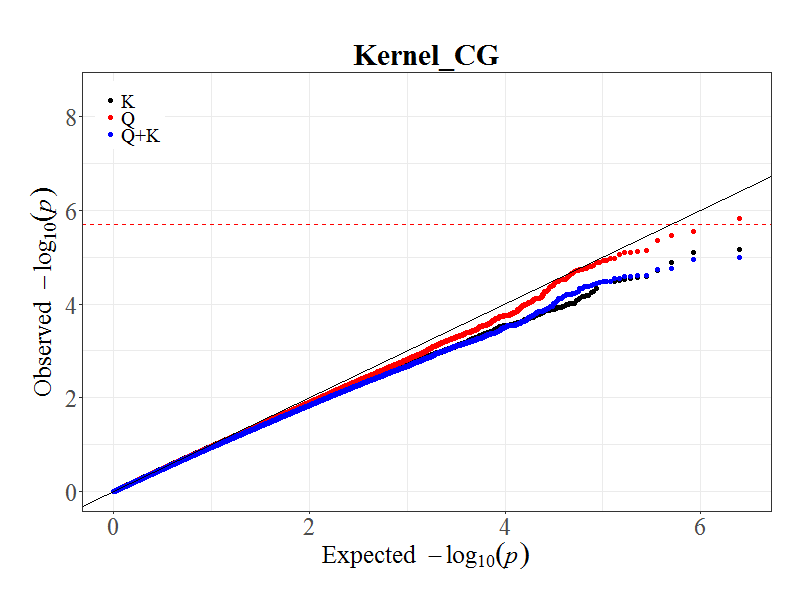

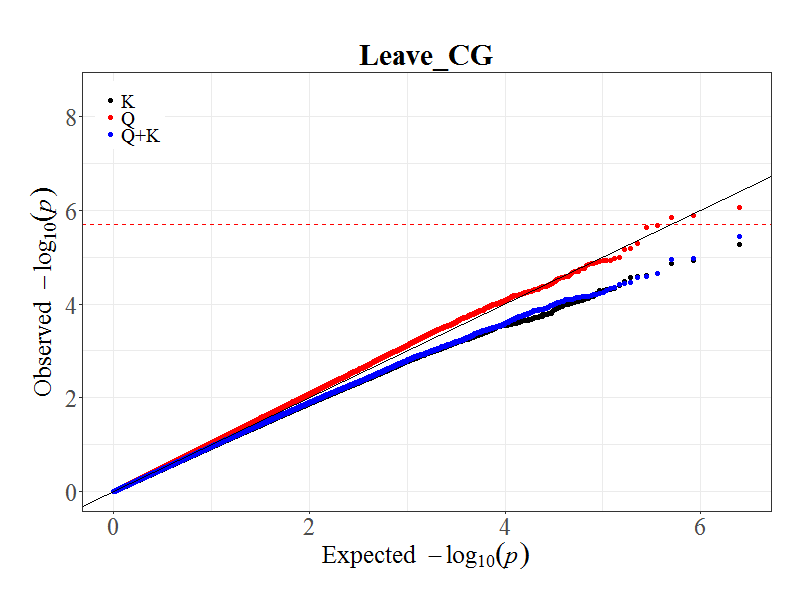

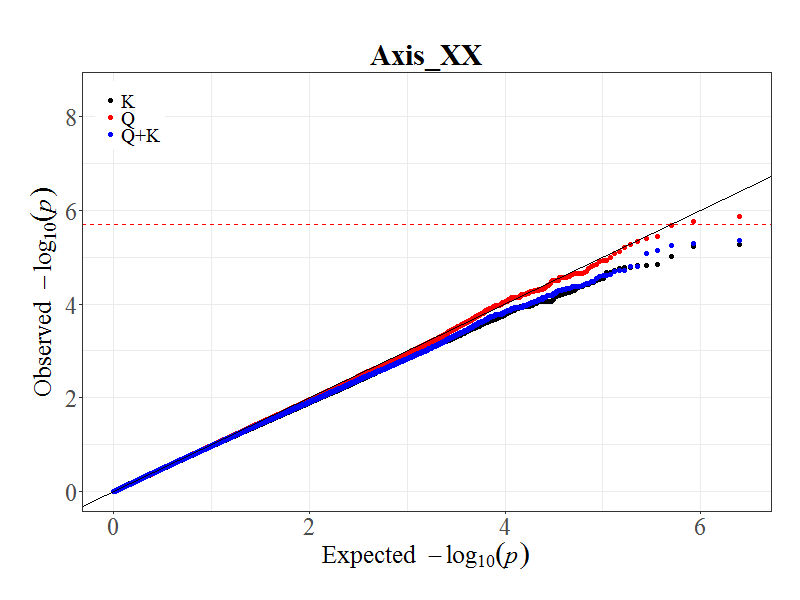

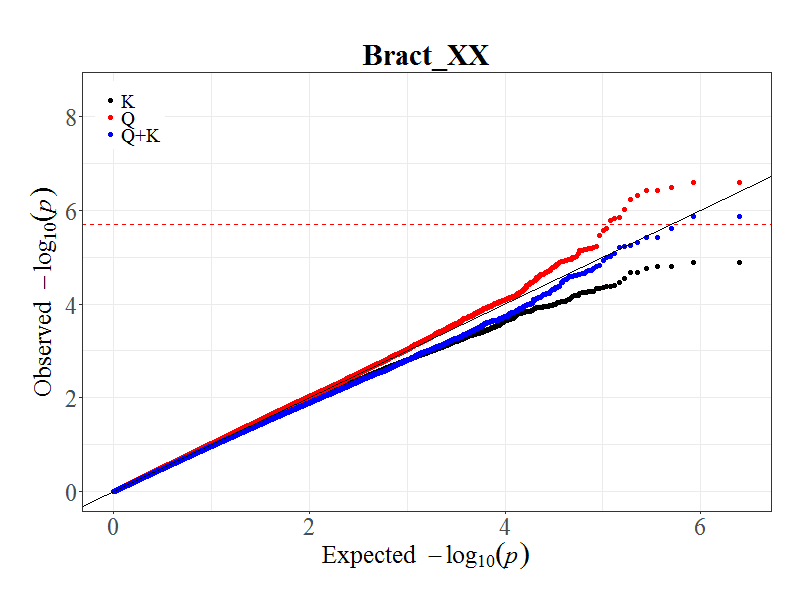

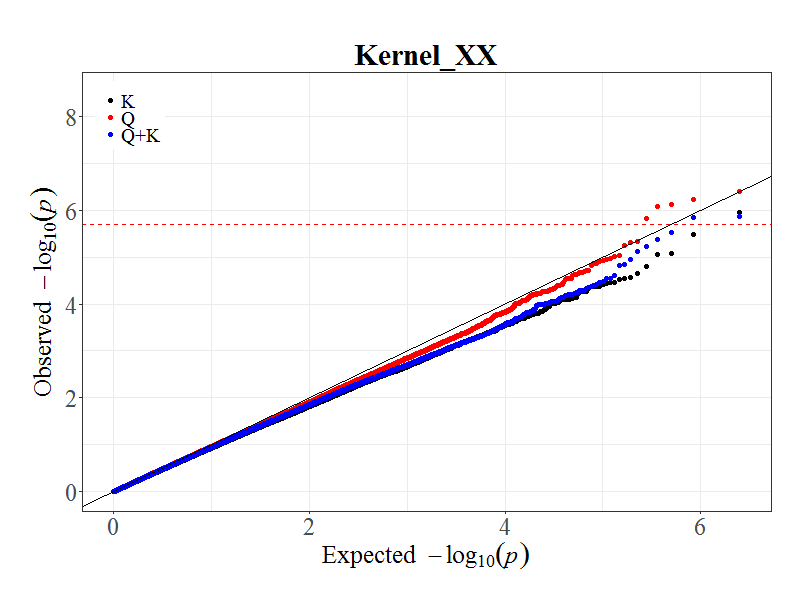

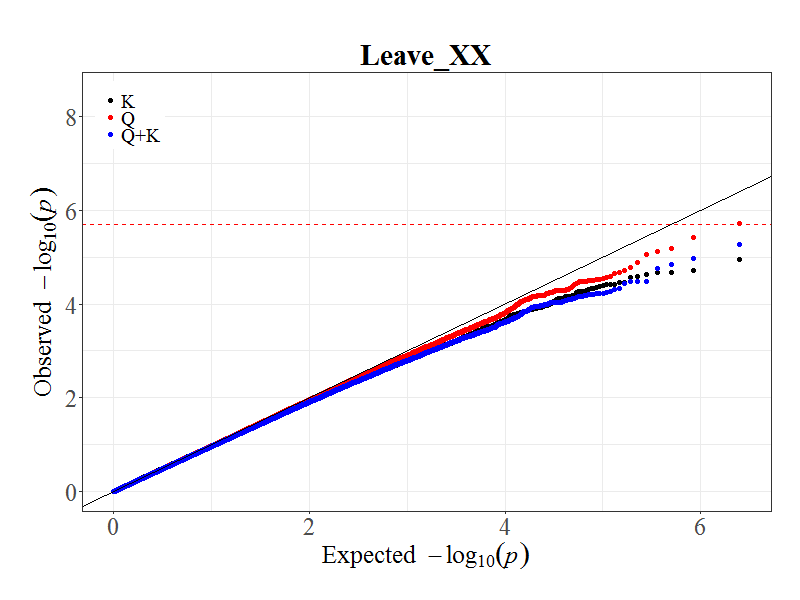

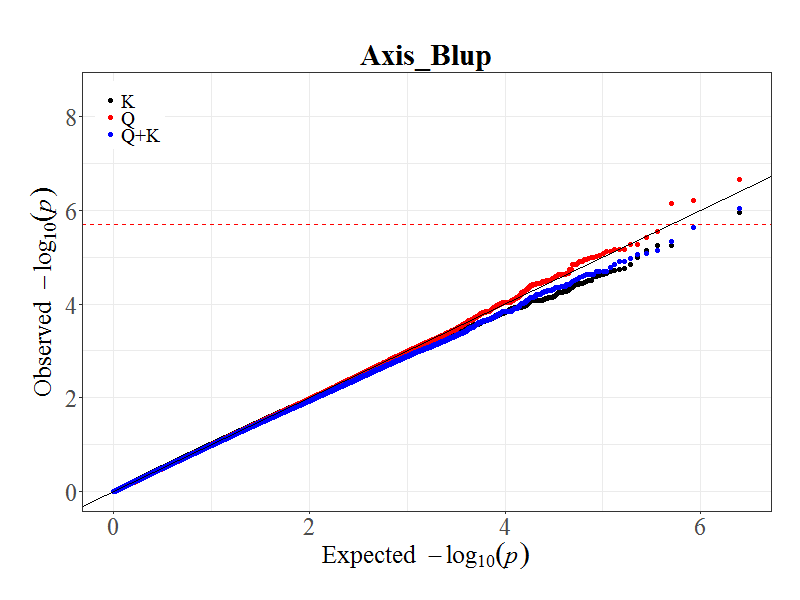

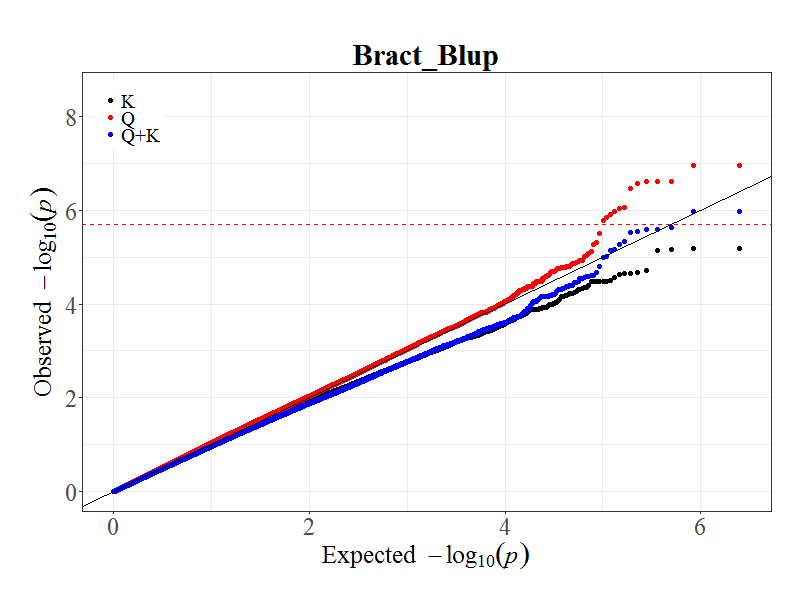

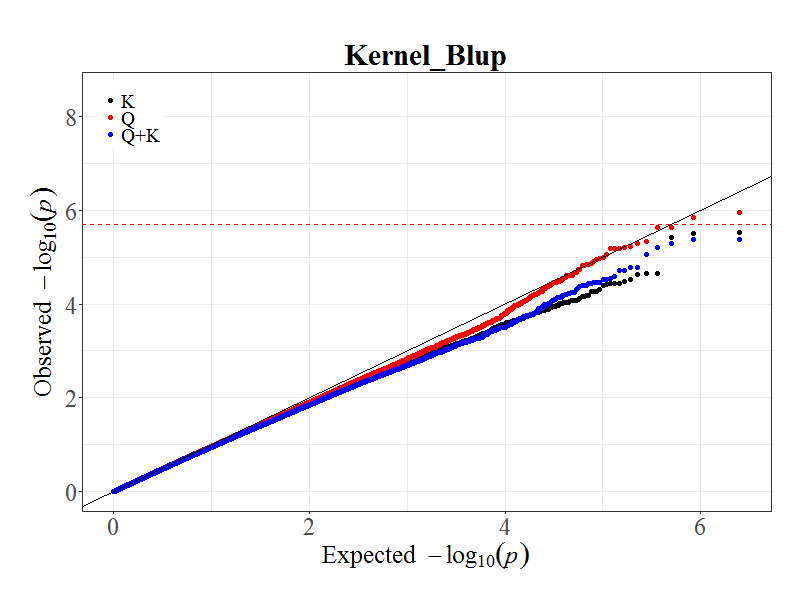

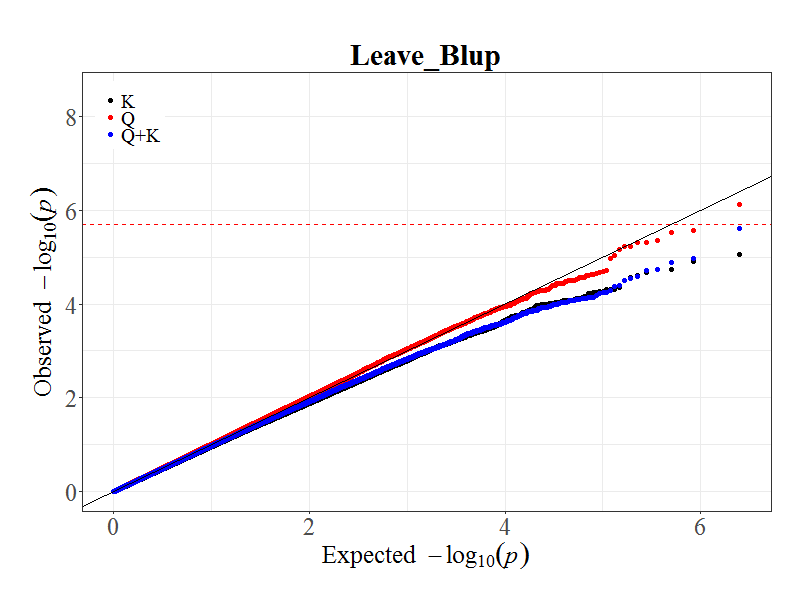

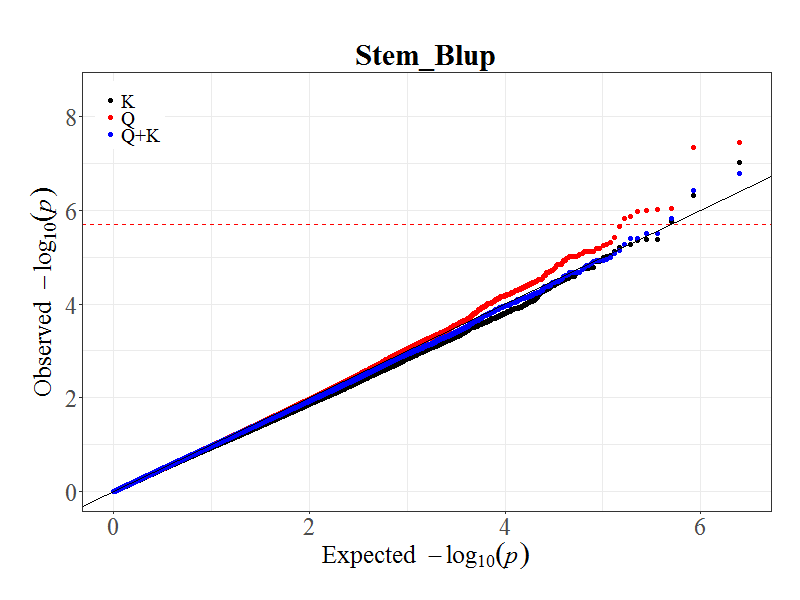


**Figure S2.** Quantile-quantile plots resulting from genome-wide association study results using three methods (Q model, K model and Q+K model) for arsenic contents in five tissues across different locations. The dashed horizontal line depicts the bonferroni-adjusted significance threshold (2.04×10^-6^).


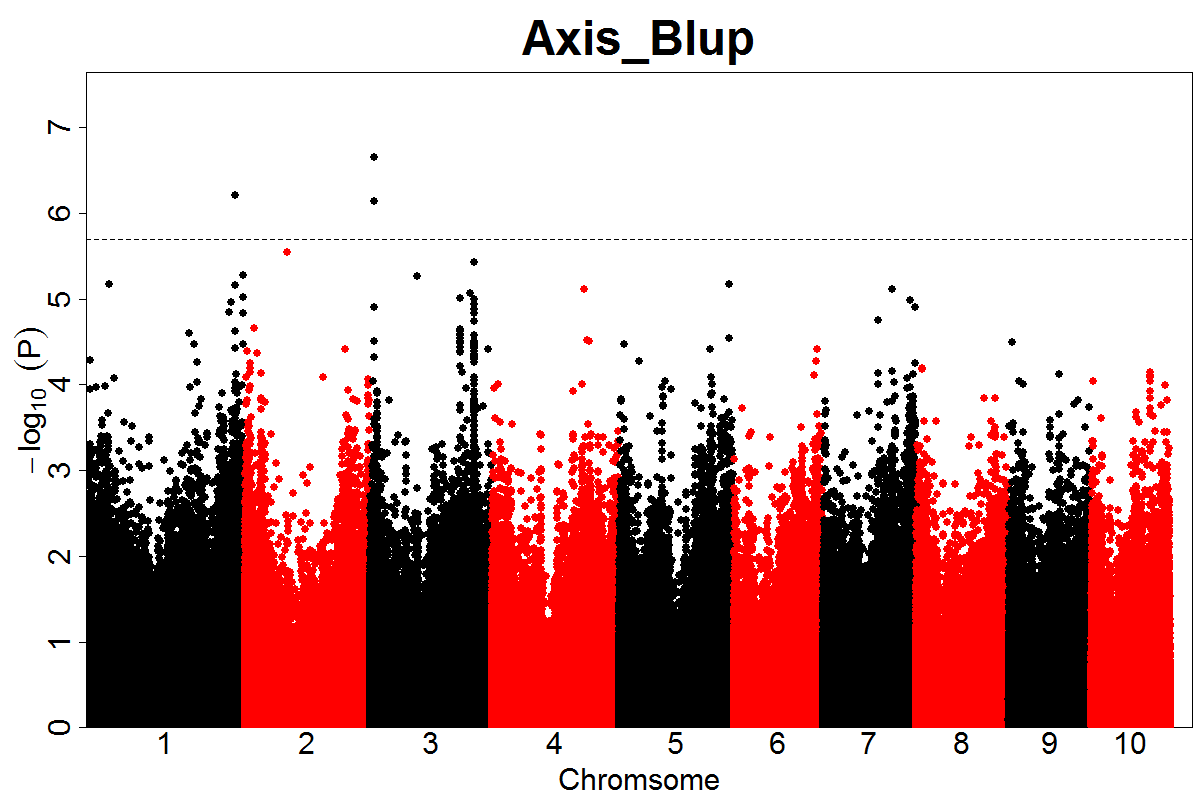

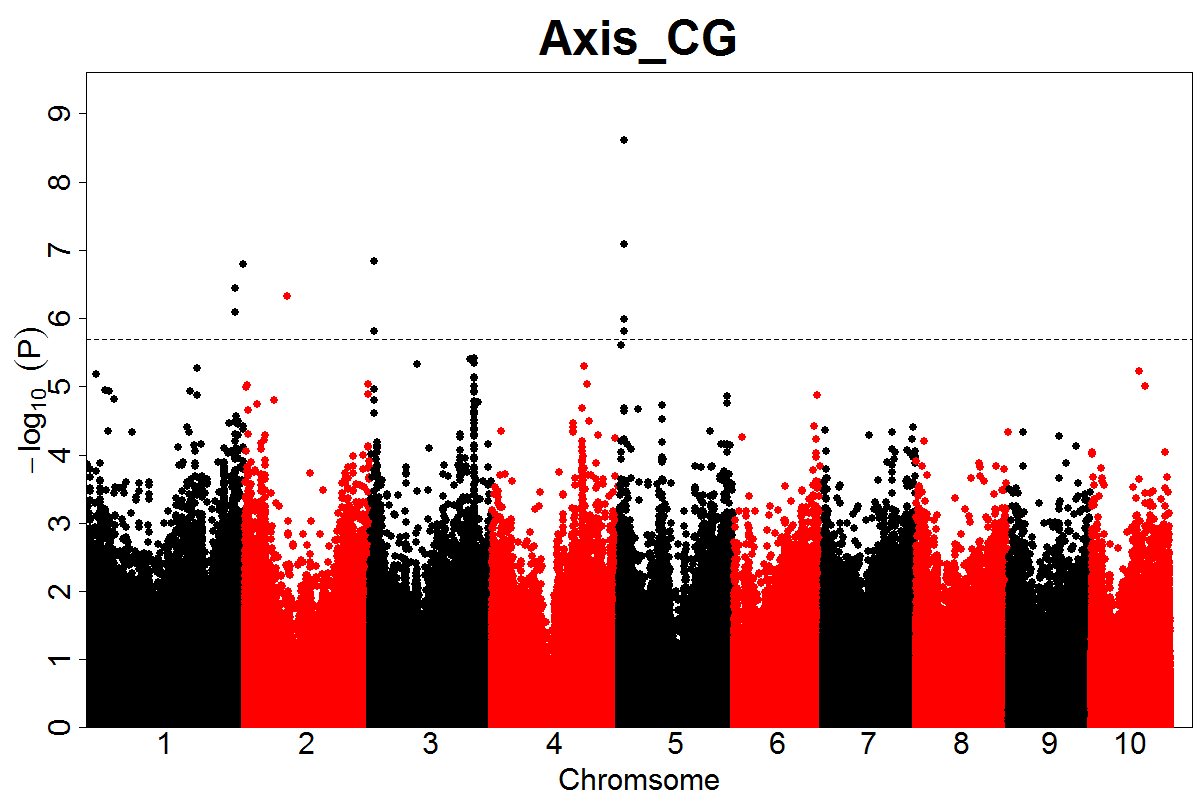

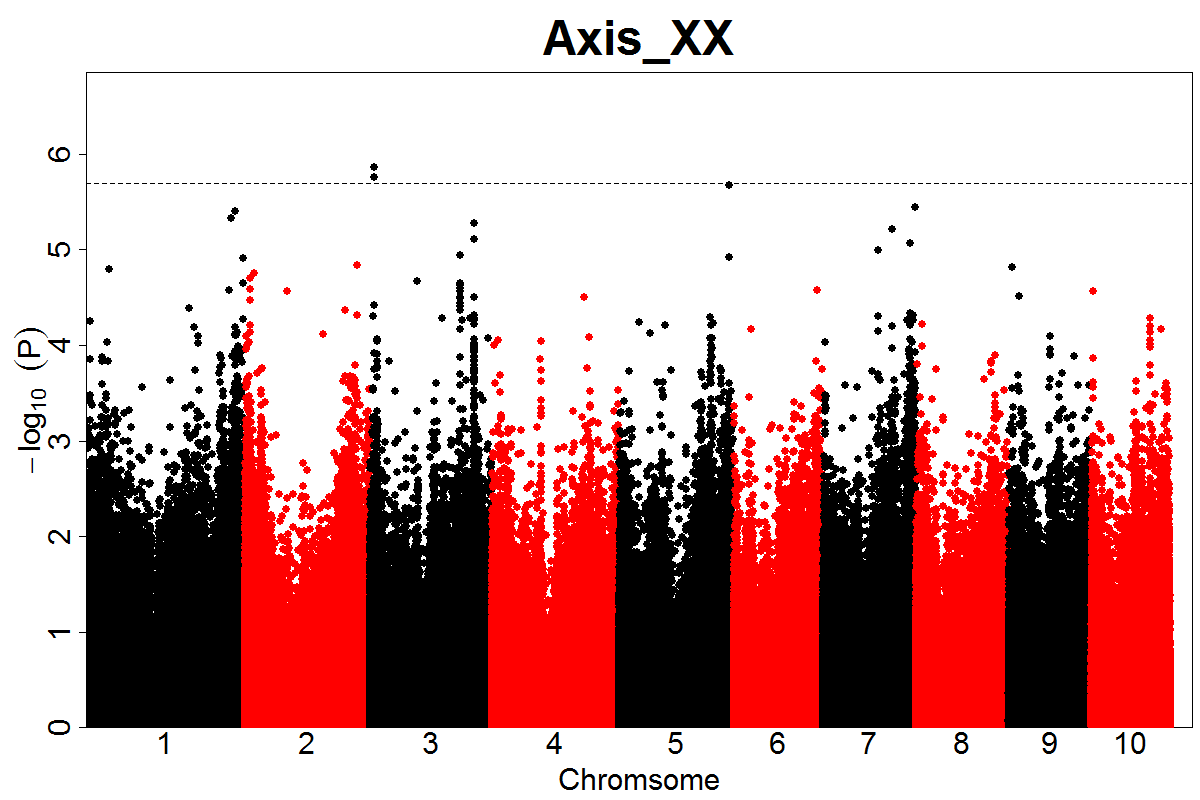

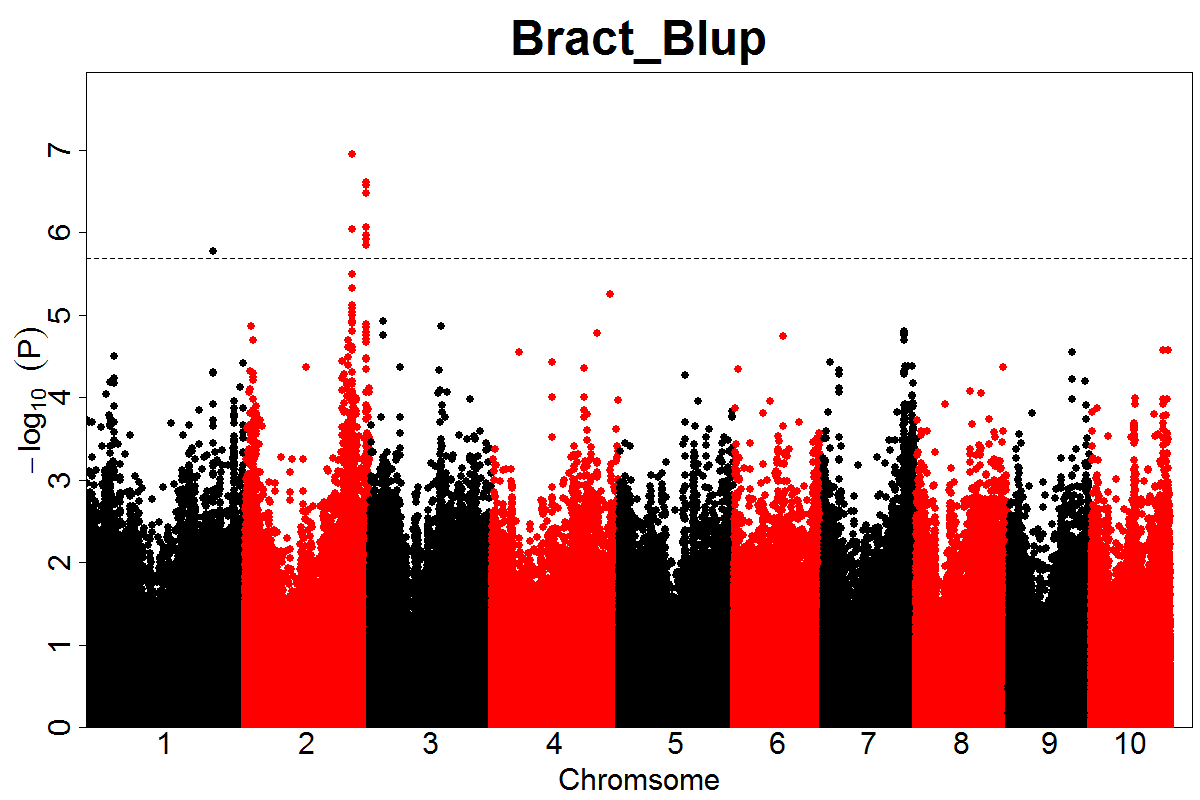

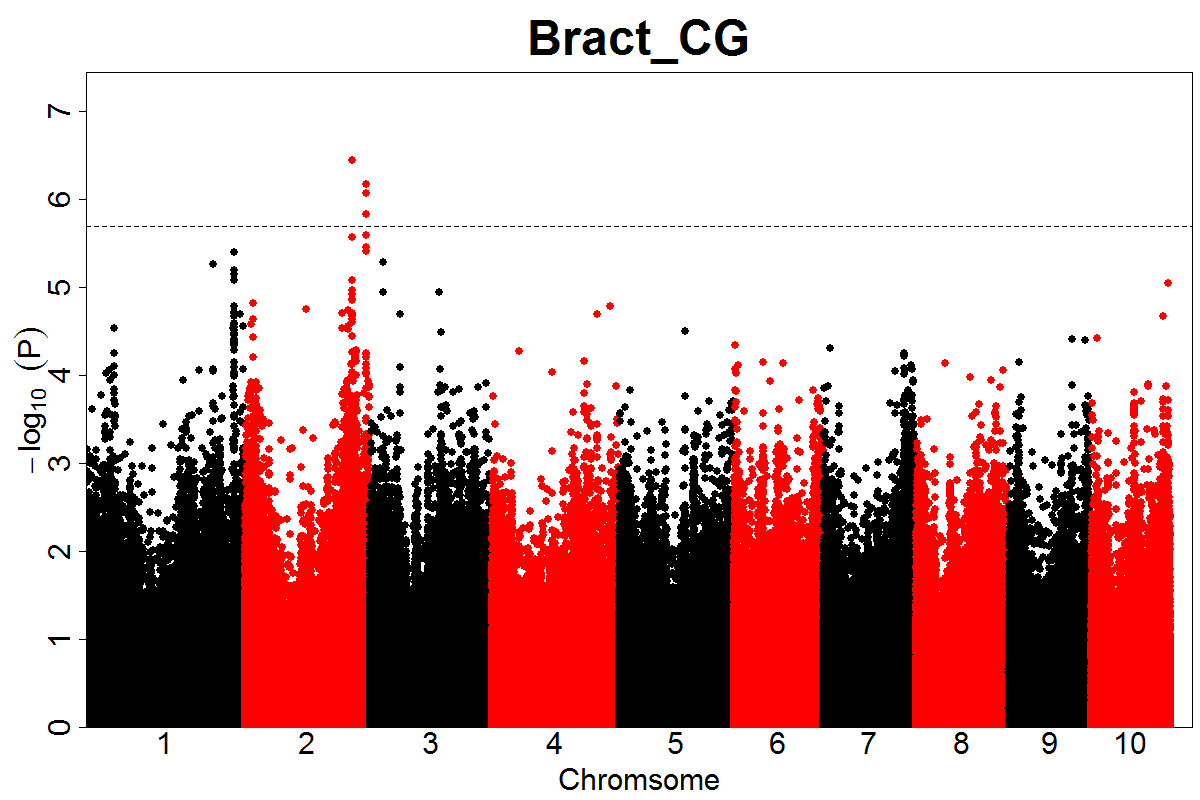

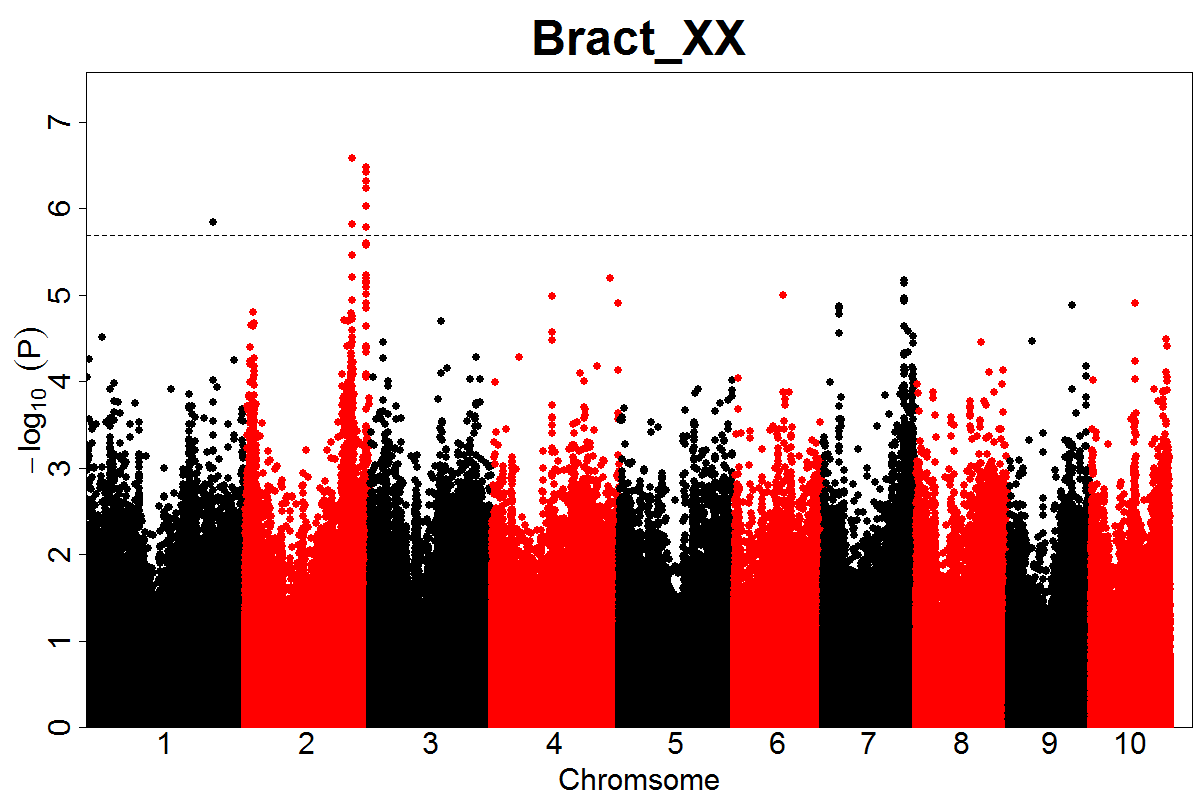

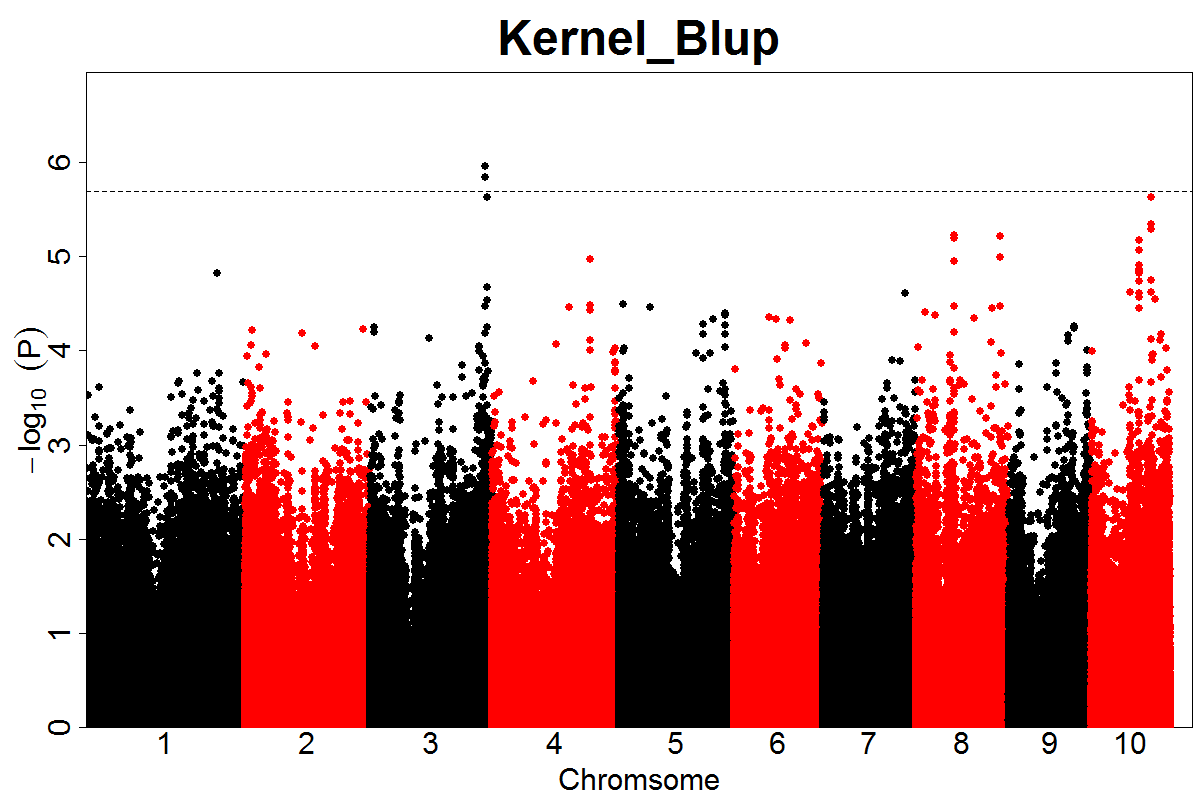

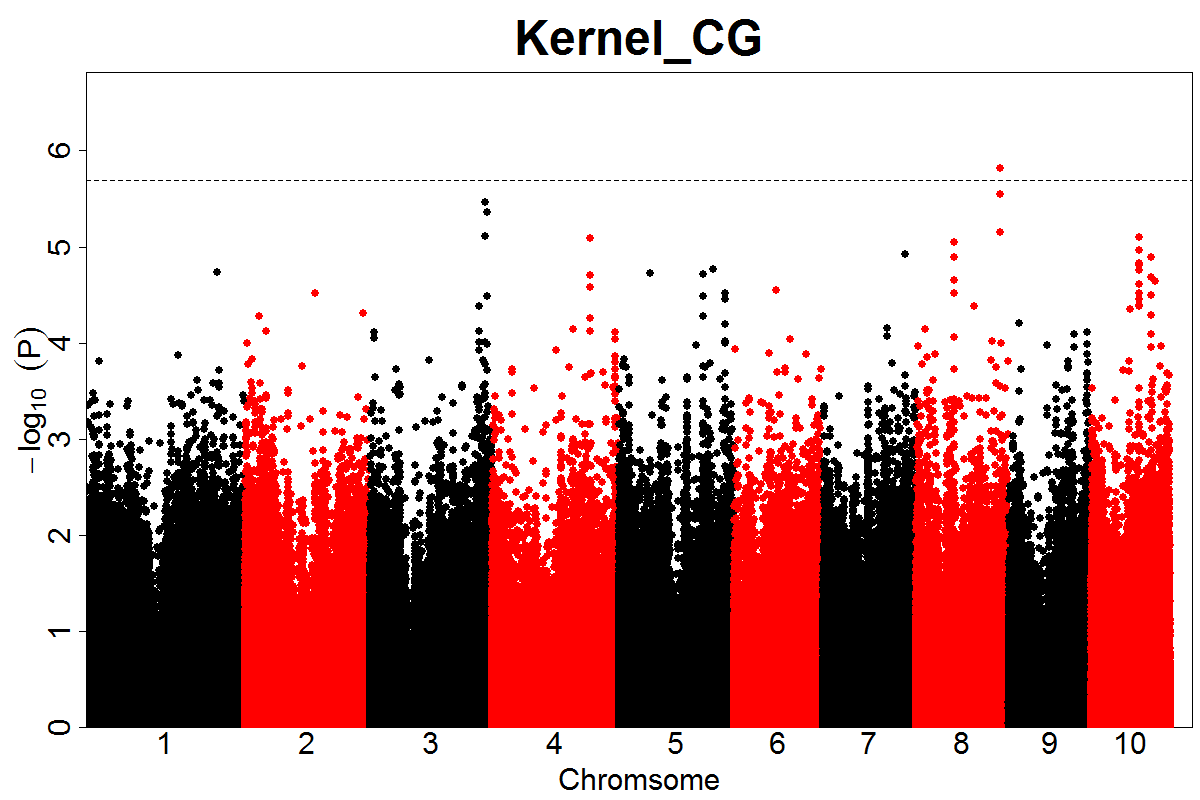

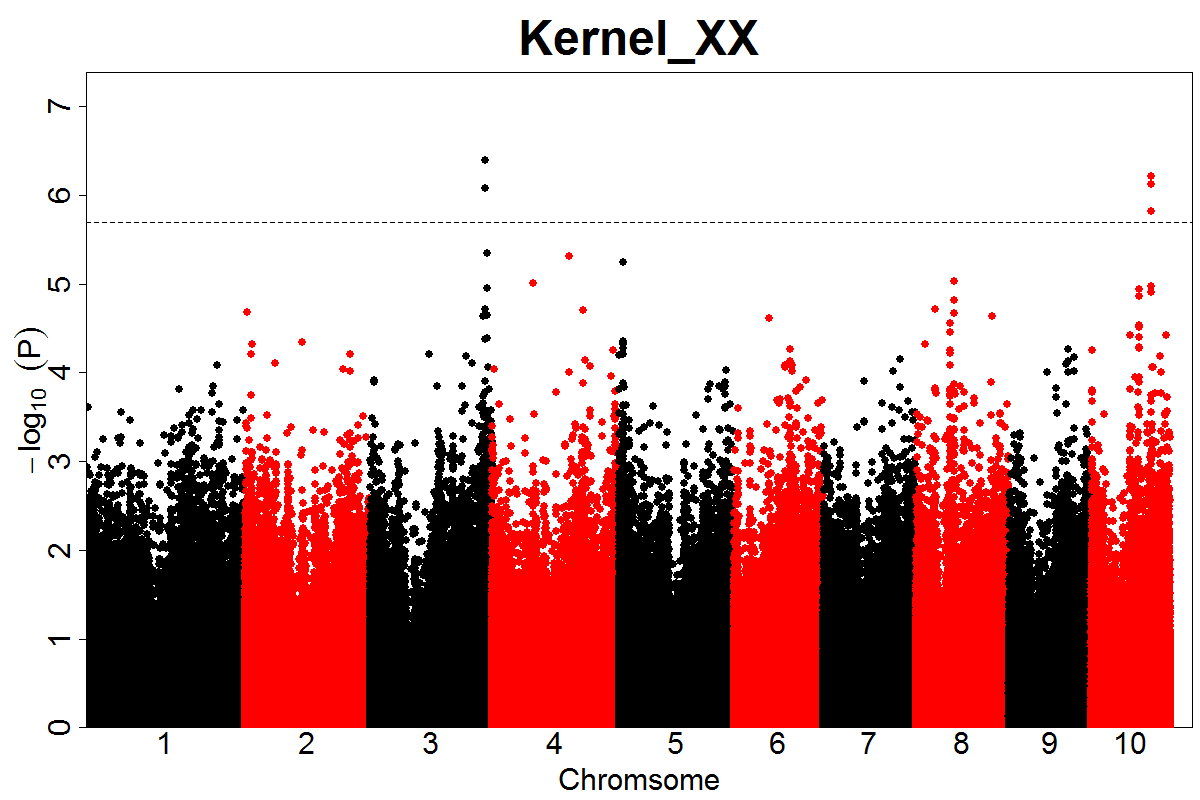

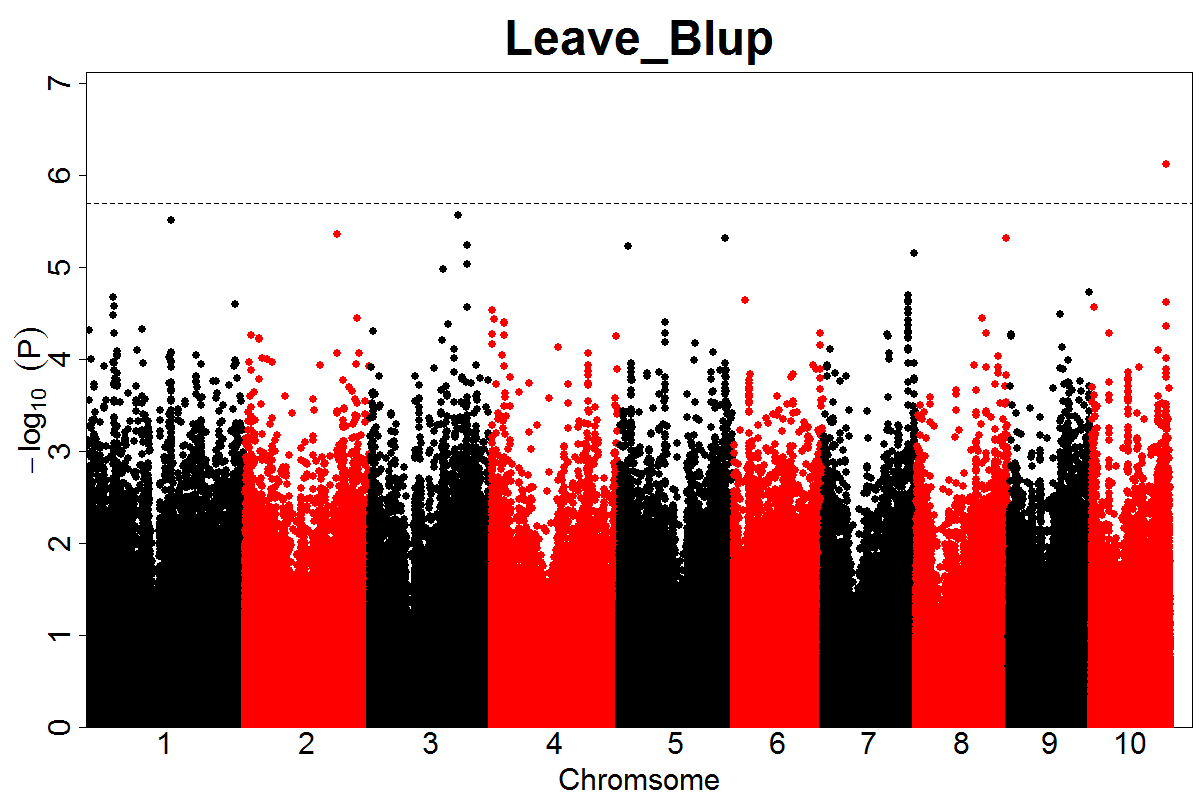

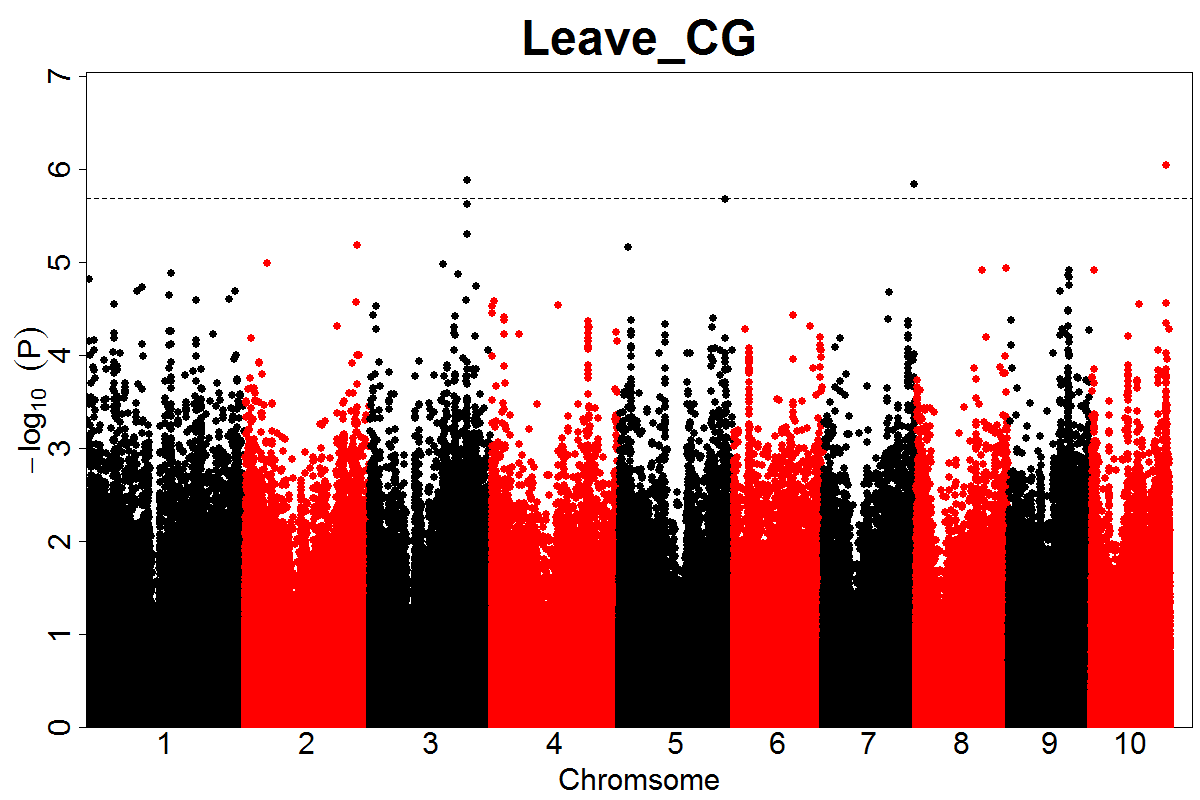

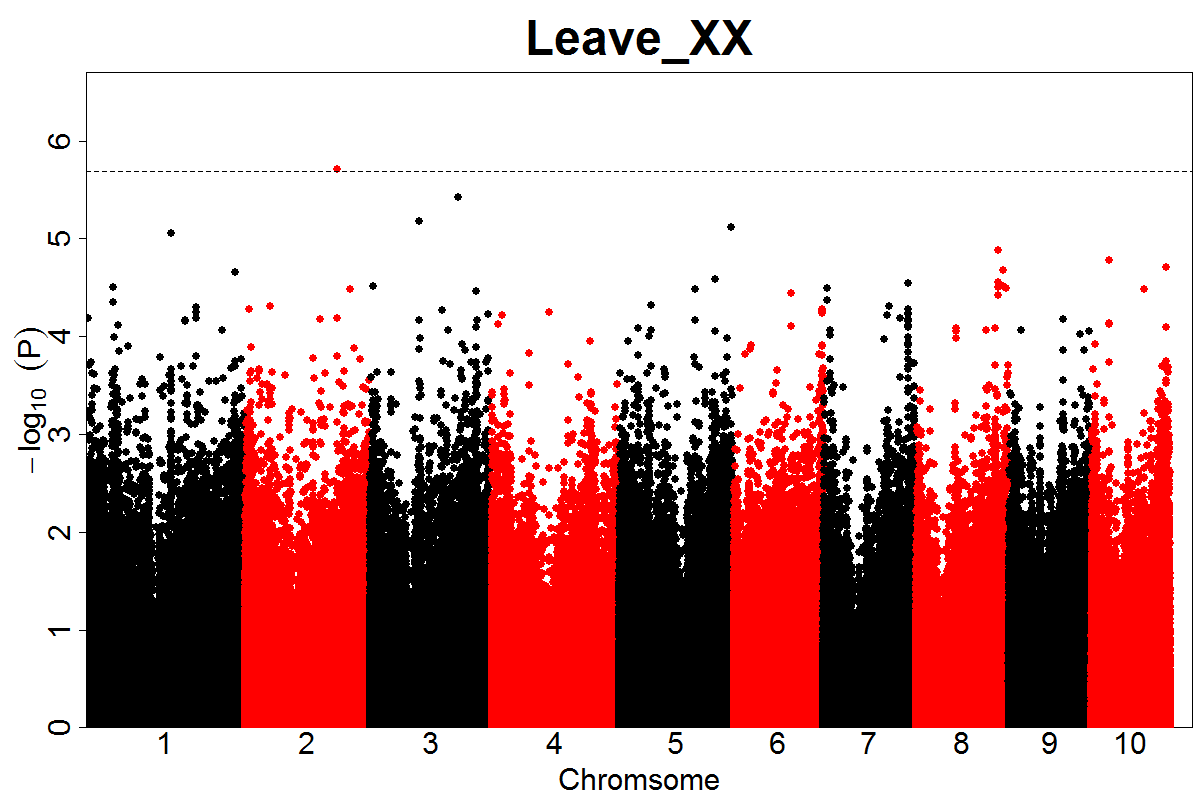

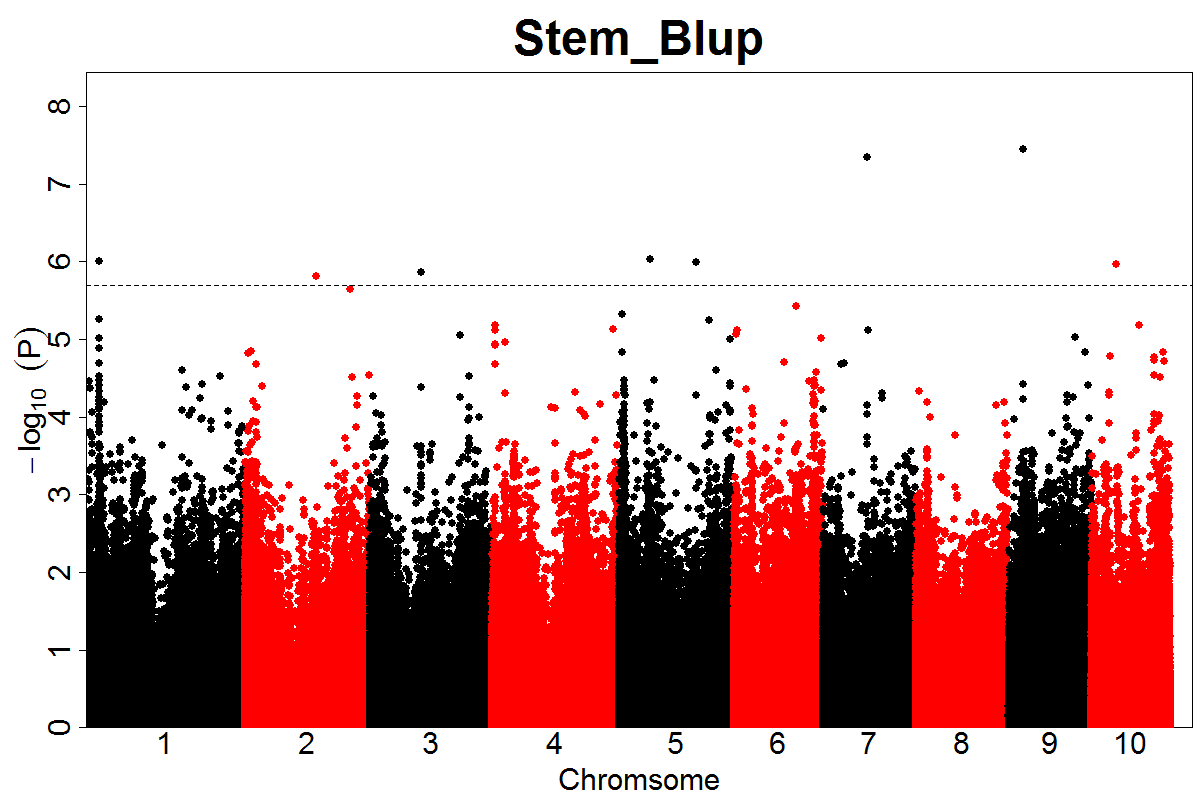

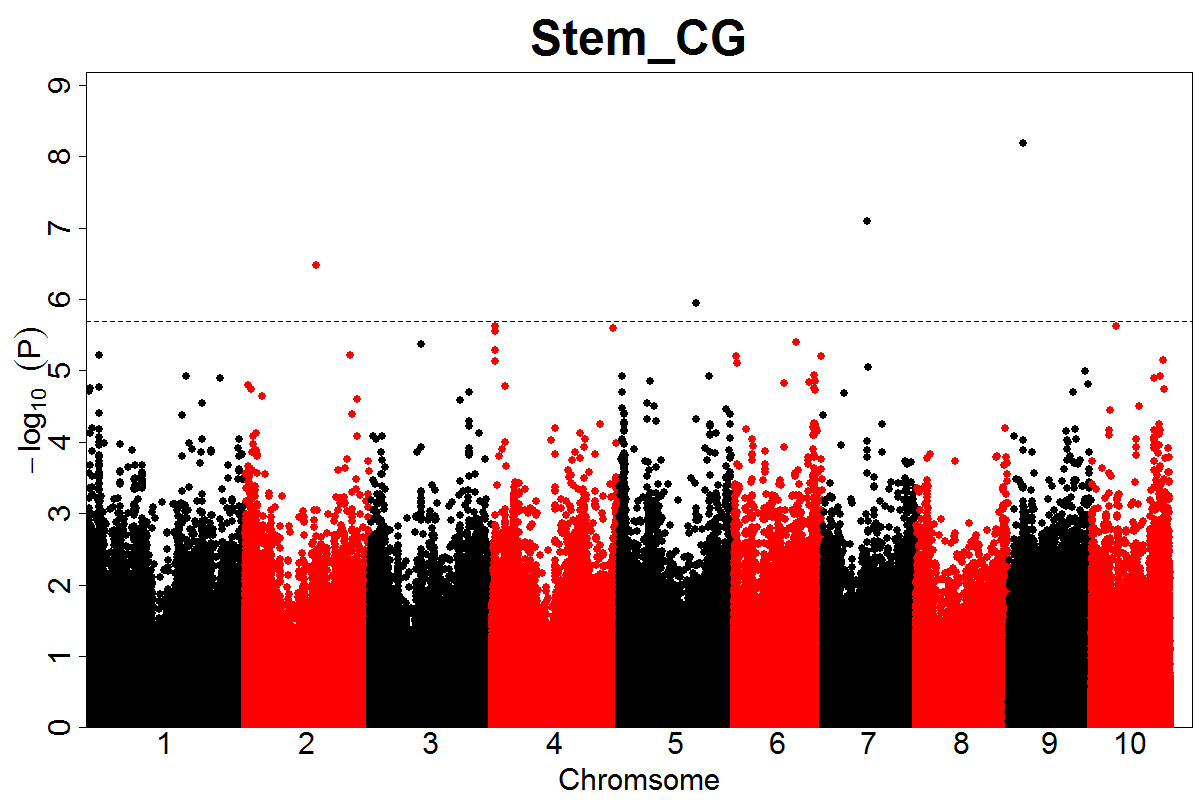

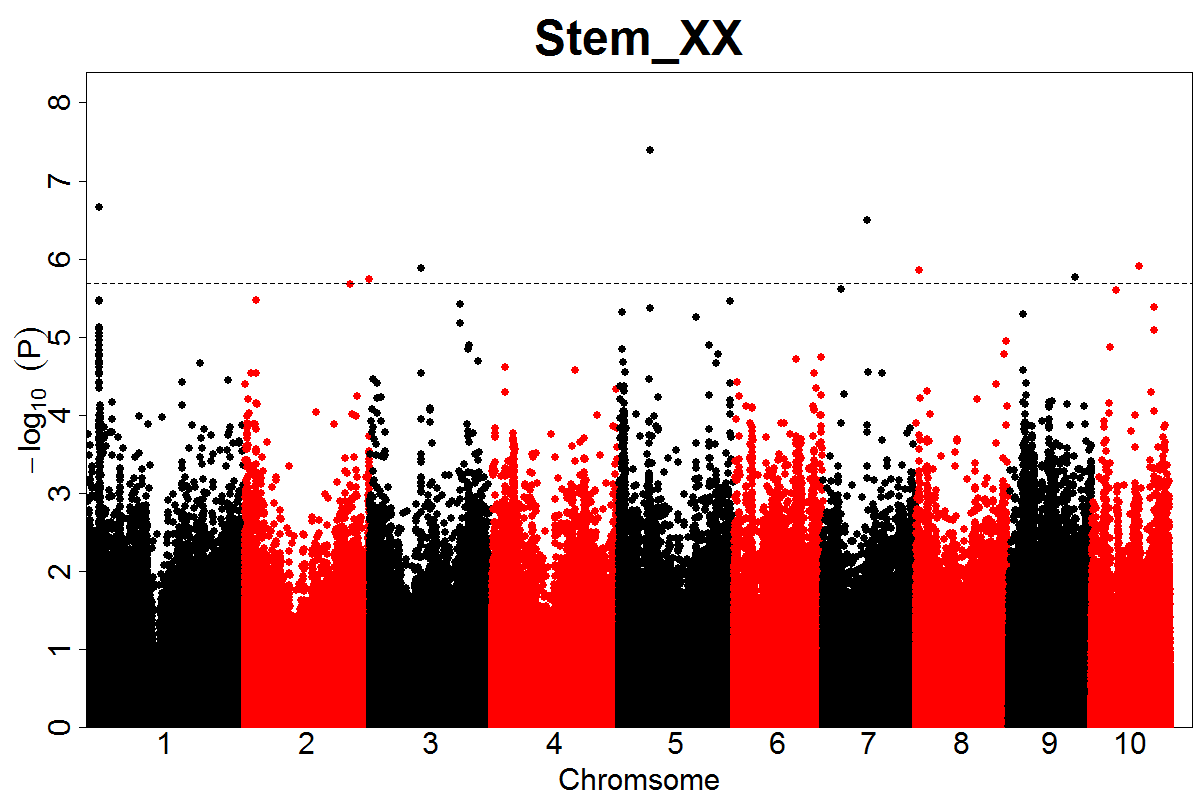


**Figure S3.** Manhattan plots for arsenic contents in five different tissues across different locations. The dashed horizontal line depicts the bonferroni-adjusted significance threshold (2.04×10-6).
